# Computational analysis of treatment resistant cancer cells

**DOI:** 10.3389/fcell.2025.1723251

**Published:** 2026-08-25

**Authors:** Alexandre Matov

**Affiliations:** DataSet Analysis LLC, San Francisco, CA, United States

**Keywords:** breast cancer type 1 susceptibility protein, circular hough transform, microtubule dynamics, taxane resistance, tubulin bundling, tubulin tyrosination

## Abstract

**Introduction:**

Prostate cancer (PC), which is a disease driven by the activity of the androgen receptor (AR), is the most commonly diagnosed malignancy and despite advances in diagnostic and treatment strategies, PC is the second most common cause of cancer mortality in men. Taxane-based chemotherapy is the only chemotherapy that prolongs survival in metastatic PC patients. At the cellular level, taxanes bind to and stabilize microtubules (MTs), inhibiting all MT-dependent intracellular pathways. MTs are highly dynamic polymers that stochastically switch between phases of growth, shrinkage, and pause. Altered MT dynamics endow cancer cells with both survival and migratory advantages. Taxanes inhibit MT dynamics and alter the spatial organization of the MT network, thereby inhibiting intracellular trafficking of molecular cargo critical for tumor survival. In PC specifically, taxanes inhibit transcriptional activity downstream of MT stabilization and AR nuclear accumulation.

**Methods:**

Different tubulin inhibitors, even from within the same structural class as the taxanes, affect distinct parameters of MT dynamics, yet the selection of taxane for chemotherapy is not based on the particular patterns of dynamic behavior of the MT cytoskeleton in individual patients. We envisage that systematic characterization using quantitative analysis of MT dynamics in PC patient cells expressing clinically relevant protein isoforms, before and after treatment with each of the taxanes, will allow us to identify criteria for the selection of the most suitable drug combination at the onset of treatment.

**Results:**

We link MT dynamics in the presence of AR variants and sensitivity/resistance to taxanes and connect fundamental research with clinically relevant concepts to elucidate cellular mechanisms of clinical response to taxanes and, thus, advance the customization of therapy. Our computational live-cell analysis addresses questions in the context of the inherent differences in MT homeostasis as a function of AR content in PC cells, the specific parameters of MT dynamics each of the taxanes affects, and how this information can be used to match endogenous patterns of MT dynamics with drug-modulated MT behavior.

**Conclusion:**

We investigate whether the sensitivity to taxanes, evaluated by computational analysis of MTs, can be linked to gene expression of the AR and its variants, whether the resistance to taxanes can be linked to the presence of a specific AR splice variant, and whether we can identify which of the taxanes will be most effective based on the endogenous patterns of MT dynamics.

## Introduction

Though treatment options for metastatic castration-resistant prostate cancer (mCRPC) have expanded over the past decade, highly proliferative phenotypes frequently emerge at the time of progression on androgen signaling inhibitors. Though initially effective in reducing tumor burden for some patients, resistance to systemic therapy is universal and approximately one-third of tumors are primarily refractory to this treatment approach. This is clinically highly significant as mCRPC refractory to tubulin inhibitors is uniformly fatal within 12-18 months (Francini et al., 2019). Currently, mCRPC chemotherapy is limited to the FDA-approved docetaxel and cabazitaxel, and could be potentially impactful (Petrylak et al., 2004; Tannock et al., 2004). However, even for the FDA-approved taxanes, there is currently no good mechanistic understanding of drug action and in mCRPC, for instance, docetaxel is always used as a first-line therapy without having to examine patient cells *ex vivo* for susceptibility (Antonarakis et al., 2017). For patients with progressive mCRPC that was refractory to AR pathway inhibitors and had received or been deemed ineligible for taxane chemotherapy, there are not many treatment options available. For patients with mutated BRCA1/2, treatment with olaparib offers a benefit (De Santis et al., 2024) and novel therapies are needed to extend survival (Bray et al., 2018; Mitchison, 2012).

AR wild type (AR-wt) and AR splice variants differentially associate with MT polymers and MT dynamics regulate AR signaling in PC (Darshan et al., 2011; Zhu et al., 2010). AR binds MTs and, upon stimulation, traffics towards the nucleus and activates gene transcription (Thadani-Mulero et al., 2012). It is unlikely AR, with a size of <0.1 μm, is transported toward MTs minus tips by cytoplasmic dynein, which traffics mRNAs (about five times larger in size than AR) and much larger molecular cargos, but rather via diffusion. In addition to AR-wt, AR splice variants are co-expressed in metastatic PC patients, such as ARv567 and AR-V7, which arise following castration (Guo et al., 2009; Hu et al., 2009; Sun et al., 2010; Watson et al., 2010). These variants, which lack the ligand-binding domain (but have the DNA-binding and transactivation domains), are insensitive to androgen deprivation therapy (ADT), and are constitutively active in the nucleus, which allows for continuous AR transcriptional activity. The ARv567 variant was shown to be present in 59% of patients with metastatic PC and to arise in response to abiraterone (Mostaghel et al., 2011). A MT co-sedimentation assay using cells transfected with the two clinically relevant AR variants, ARv567 and ARv7, showed that the majority of ARv567 is associated with MTs, while only 40% of ARv7 co-fractionated with MT polymers (Thadani-Mulero et al., 2014). Further, paclitaxel treatment inhibited ARv567 nuclear accumulation, while it had no effect on variant ARv7 (Thadani-Mulero et al., 2014). Importantly, the lack of inhibition of ARv7 nuclear accumulation is correlated with docetaxel resistance *in vivo* in a LuCAP xenograft model of PC (Nguyen et al., 2017). Furthermore, these *in vivo* data showed that the ARv567 variant is very sensitive to docetaxel treatment and the high level of sensitivity to docetaxel remains persistent for 26 weeks of treatment (Nguyen et al., 2017). Taken together, these data indicate a dependency of AR’s transcriptional activity on the dynamics of the MT cytoskeleton.

## Results

### Computational analysis of MT dynamics

Our computational approach for the analysis of MT dynamics (Jordan and Wilson, 2004) in living cells is based on the automated tracking of the motion of MT growing (or plus) tips in high-resolution confocal microscopy image sequences using a fluorescently tagged MT plus-tip marker EB1 or EB3 (end-binding protein 1 or 3) (Matov et al., 2010). EB1 and EB3 recognize conformational changes and bind the MT plus tips during polymerization (growth) (Slep and Vale, 2007). When EB1 (or EB3) is EGPF-labeled, the EB1ΔC-2xEGFP dimers at the MT plus tip form a fluorescent comet, which serves as a molecular marker for the direct visualization of MT polymerization dynamics (Akhmanova and Steinmetz, 2008). Our image analysis algorithm tracks thousands of EB1 comets visible in an image time-lapse sequence, allowing the detection of spatial patterns of MT dynamics and introducing spatiotemporal clustering of EB1ΔC-2xEGFP growth tracks to infer MT behaviors during phases of pause and depolymerization (shrinkage/shortening). EB1 comet detection is accomplished by band-pass filtering implemented as a Difference of Gaussians (Wilson and Giese, 1977) operator (for comparisons with a Laplacian of Gaussian and the Harris operator, see (Lindeberg, 2014)), which enhances image features with the expected shape and size of an EB1 comet, while suppressing higher-frequency imaging noise and lower-frequency structures representing larger than the EB1 comets aggregates of non-specific fluorescently labeled proteins. We compute the sum of the squared intensity differences between each segmented EB1 comet and the average comet profile to exclude the few detections with unusual shapes due to labeling artifacts via unimodal histogram thresholding (Rosin, 2001). Our EB1 comet detection algorithm is robust against variations in comet lengths and brightness, both within videos and between videos. The feature detector delivers the position of each comet in a time-frame as well as the eccentricity and angular orientation of the comets. The latter is used as a directional cue in the subsequent motion tracking of comets from one time-frame to the next.

The cost of linking comets is defined in statistical terms as the Mahalanobis distance between projected and extracted comets (Mahalanobis, 1936). Detected EB1 comets are then tracked using a linear Kalman (Swerling, 1959) filter-based algorithm (Yang et al., 2005). The EB1 comets serve as a molecular marker for the direct visualization of MT polymerization dynamics only and we introduced spatiotemporal clustering of EB1ΔC-2xEGFP growth tracks to infer MT behaviors during the phases of pause and depolymerization. Clustering of collinear MT growth tracks that likely belong to the same MT is performed by solving the bipartite graph as a linear assignment problem (LAP) via the Hungarian algorithm (Kuhn, 1955). It identifies the globally optimal positional correspondences between the endpoints of EB1 tracks and the start points of new tracks, and every potential assignment is characterized by a cost of linking (Matov et al., 2010). Specifically, a pairing between an EB1 track termination and a consequent track initiation is considered only if these two events are separated by less than 15s or another use-specified time-lag. Furthermore, the track initiation must fall within either one of two cone-shaped, geometrical search regions. The choice of the forward cone with an opening half-angle of 60° is motivated by the observation that during pauses or out of focus movements, MTs sometimes undergo significant lateral displacements and directional changes, especially near the cell edge. In contrast, the backward cone has an opening half-angle of 10° only, motivated by the observation that MT shortening generally follows the recent MT growth track until a MT rescue or the complete depolymerization of the MT. By construction, this track assignment is spatially and temporally global, which warrants a high level of robustness.

### Cytoskeleton regulation in PC

#### MT dynamics in M12 isogenic PC cell lines expressing GFP-tagged wt- or variant-AR

**FIGURE 1:**
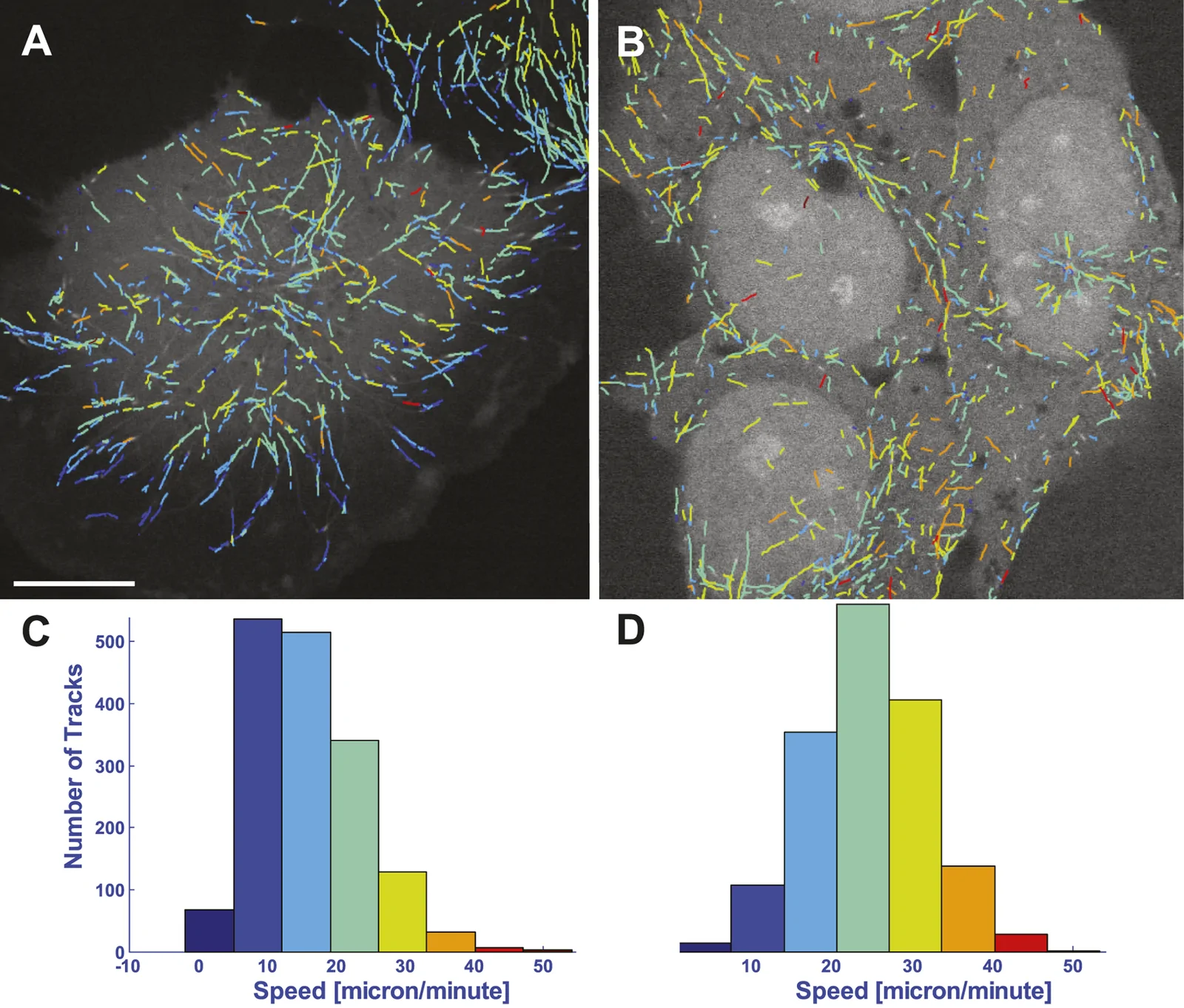
Tracking of EB1 comets in M12 isogenic PC cell lines expressing GFP-tagged wt- or variant-AR. MT tips and AR are labeled with GFP and imaged for a minute (with an acquisition rate of two images per second). EB1 comets are computationally tracked (Yang et al., 2005). The color-coding represents EB1 comet speeds; colder colors correspond to lower speeds, and warmer colors correspond to faster speeds. Scale bar equals 5 µm. **(A)** MT growth tracks for PC cells expressing AR-wt. The median speed was about 15 µm with a marked slowdown at the cell edge, where there was no AR. **(B)** MT growth tracks for cells expressing the ARv7 variant which is resistant to paclitaxel treatment. The median speeds were about 24 μm/min. The lower panels show the corresponding EB1 comet speed histograms. Histograms of MT growth speeds in µm/min are shown on **(C)** for AR-wt and **(D)** for the ARv7 variant.

We used cells from an isogenic PC cell line series of the M12 cells expressing GFP-tagged AR-wt or variant-AR and measured MT polymerization rates in three cell lines of the M12 series – parental cells with no endogenous AR, AR-wt cells, and ARv7 variant cells – which simultaneously express EB1ΔC-2xEGFP. In metastatic PC patients, the treatment of tumors which express the ARv7 variant correlates with clinical taxane resistance (Hörnberg et al., 2011). Also, increased MT dynamicity has been correlated with taxane resistance (Gonçalves et al., 2001). Figure 1 shows MT growth tracks (EB1 comet tracks) overlaid on raw image data from M12 cells stably expressing AR-wt (Figure 1A) or ARv7 (Figure 1B). In cells expressing the AR-wt, in comparison to parental cells not expressing AR, MT dynamicity did not change drastically (e.g., the average speed of EB1 comets was reduced to 16.2 μm/min (Figure 1C) from 17.3 μm/min (Supplementary Figure S1) in the AR-null cells). However, when we expressed the ARv7 variant, EB1 comet speeds increased by over 40% to an average of 24.6 μm/min (Figure 1D). In M12 AR-wt cells, AR is localized both in the cytoplasm and the nucleus (Figure 1A, the overall “haze” visible in the background of the images is a result of the fluorescent AR). In M12 ARv7 cells, AR localization was predominantly nuclear (Figure 1B, see the brighter nuclear areas), in agreement with published data (Guo et al., 2009). Additionally, in cells expressing AR-wt, EB1 comet speeds slow down considerably as MTs approach the cell edge (Figure 1A), while no such slowdown was observed in M12 ARv7 cells (Figure 1B). We also clearly observed differential spatial organizations of the MT cytoskeleton, with M12 ARv7 cells’ EB1 comet tracks rarely overlapping with the nuclear areas. Together, the results indicate differential regulation in taxane-sensitive and taxane-resistant cells.

#### Patient-derived organoids

Localized PC can be cured through surgical intervention. However, for the patients who are not good candidates for this intervention, for example, those with high risk localized tumors or with locally advanced or metastatic disease, the question of whether therapy with tubulin inhibitors would be beneficial is of utmost importance. Clinically, the use of tubulin inhibitors to treat advanced PC is expected to significantly increase based on the results of the CHAARTED (Sweeney et al., 2015) and STAMPEDE (Vale et al., 2016) studies, which showed about 14 and 22 months survival benefit, respectively, based on combination of ADT and taxane therapy. Similarly, the STAMPEDE trial showed the benefit of docetaxel for early treatment of locally advanced tumors. Approximately one-third of tumors are primarily refractory to this treatment approach, yet markers predicting drug efficacy are not available and the mechanisms of resistance are not understood. Therefore, there is an unmet clinical need to match individual tumors to a tubulin inhibitor most likely to be efficacious (Matov, 2024e). We propose to identify such matches based on MT dynamics signatures in metastatic PC organoids (Gao et al., 2014) and derived organoids from primary (UCSF-PR), micro-metastatic (UCSF-LN), and distant metastasis (UCSF-PCa) tumor tissues from 16 PC patients.

**FIGURE 2:**
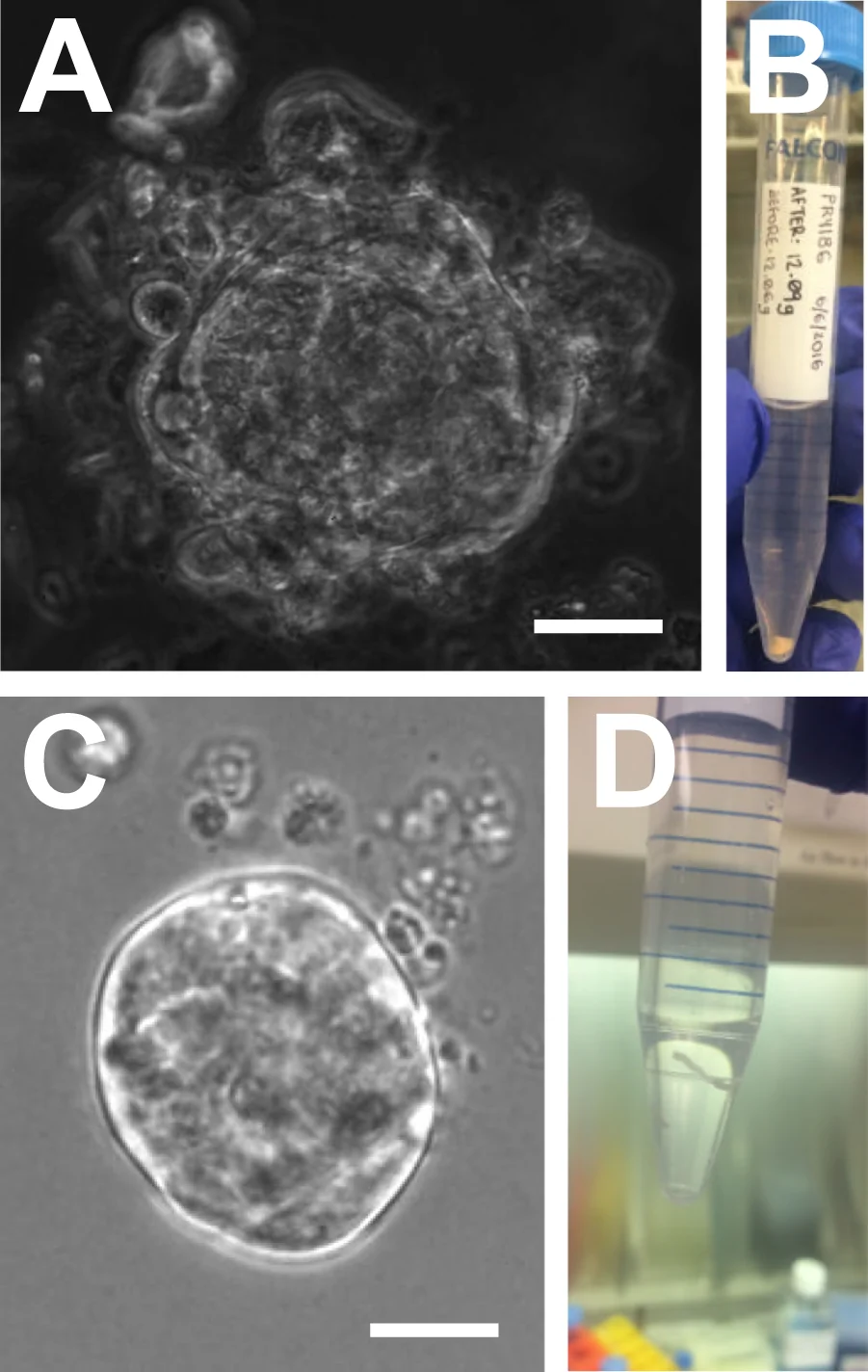
Phase contrast and transmitted light live-cell images of novel organoids. **(A)** UCSF-PR6 (primary tumor) on Day 29 after plating of single tumor cells. Scale bar equals 50 μm. **(B)** Tissue (30 mg) dissociated to derive UCSF-PR6 from 3 mm punch core biopsy. **(C)** UCSF-PCa1 (metastatic tumor, visceral tissue (liver)) organoid on Day 14 after plating of single tumor cells. Scale bar equals 30 μm. **(D)** Tissue (1 mg) dissociated to derive UCSF-PCa1 from needle biopsy of the liver.

We dissociated prostate tissues (Figure 2) according to (Goldstein et al., 2011) and cultured organoids according to (Drost et al., 2016; Gao et al., 2014). The main hurdle in culturing primary prostate tumors has been the lack of markers to select against normal tissue cells (Drost et al., 2016). In colorectal cancer organoids selection against normal cells is done by removing R-spondin1, an effector of Wnt signaling, from the medium (van de Wetering et al., 2015).

**FIGURE 3:**
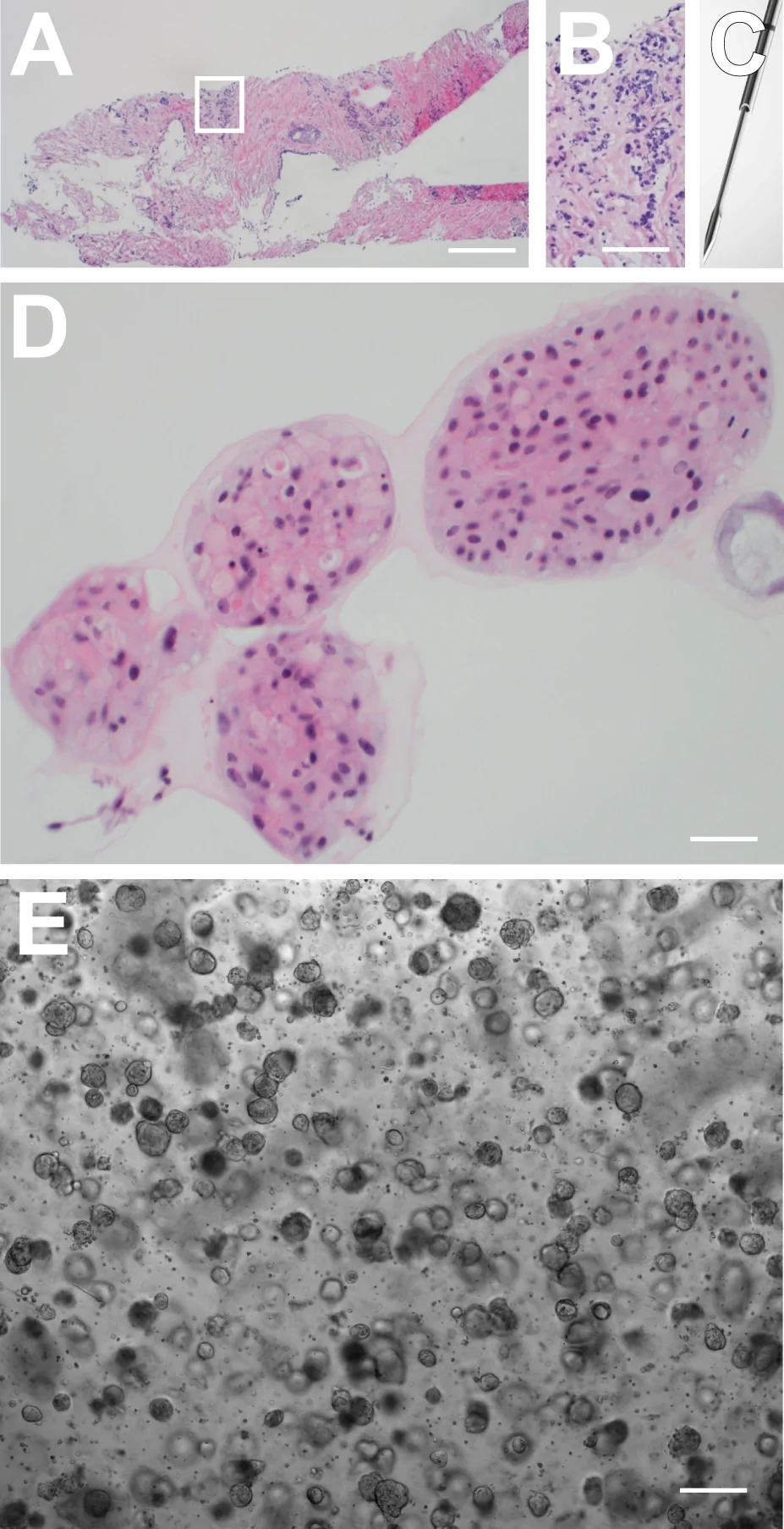
PC tissue histology, organoid culture and histology. **(A)** H&E of the tissue sample for UCSF-PR5 obtained with twin-needle biopsy, Gleason Score 5 + 3 + 4; in the white rectangle is area of high-grade tumor (Gleason Score 5). Scale bar equals 100 μm. **(B)** Enlarged zone of tumor pattern with Gleason Score 5 from the white rectangle in **(A)**. Scale bar equals 40 μm. **(C)** Twin-needle provides matching tissue pairs, one for organoid culture and one for histology (shown in (A)). **(D)** Pathology assessment determines benign organoid culture by histology of UCSF-PR4 derived from 2 mg punch core biopsy tissue with Gleason Score 4 + 3 and 10% tumor density. Scale bar equals 20 μm. **(E)** Transmitted light image of small size, round organoid from UCSF-PR7 on Day 10 after seeding of single patient cells. Scale bar equals 200 μm.

However, there is no such molecular marker in primary PC. Because we were aware of the issue of mixed tissue in primary tumors, we initially tried to minimize the amount of the sample by using a specialized twin-needle (Figure 3) to increase the likelihood of capturing the tumor tissue only as well as to have a matching tissue for histology. However, we found tissue with Gleason Score 5, 4, 3 as well as normal tissue within less than 1 mg sample (Figure 3).

#### Y-27632 ROCK inhibitor

**FIGURE 4:**
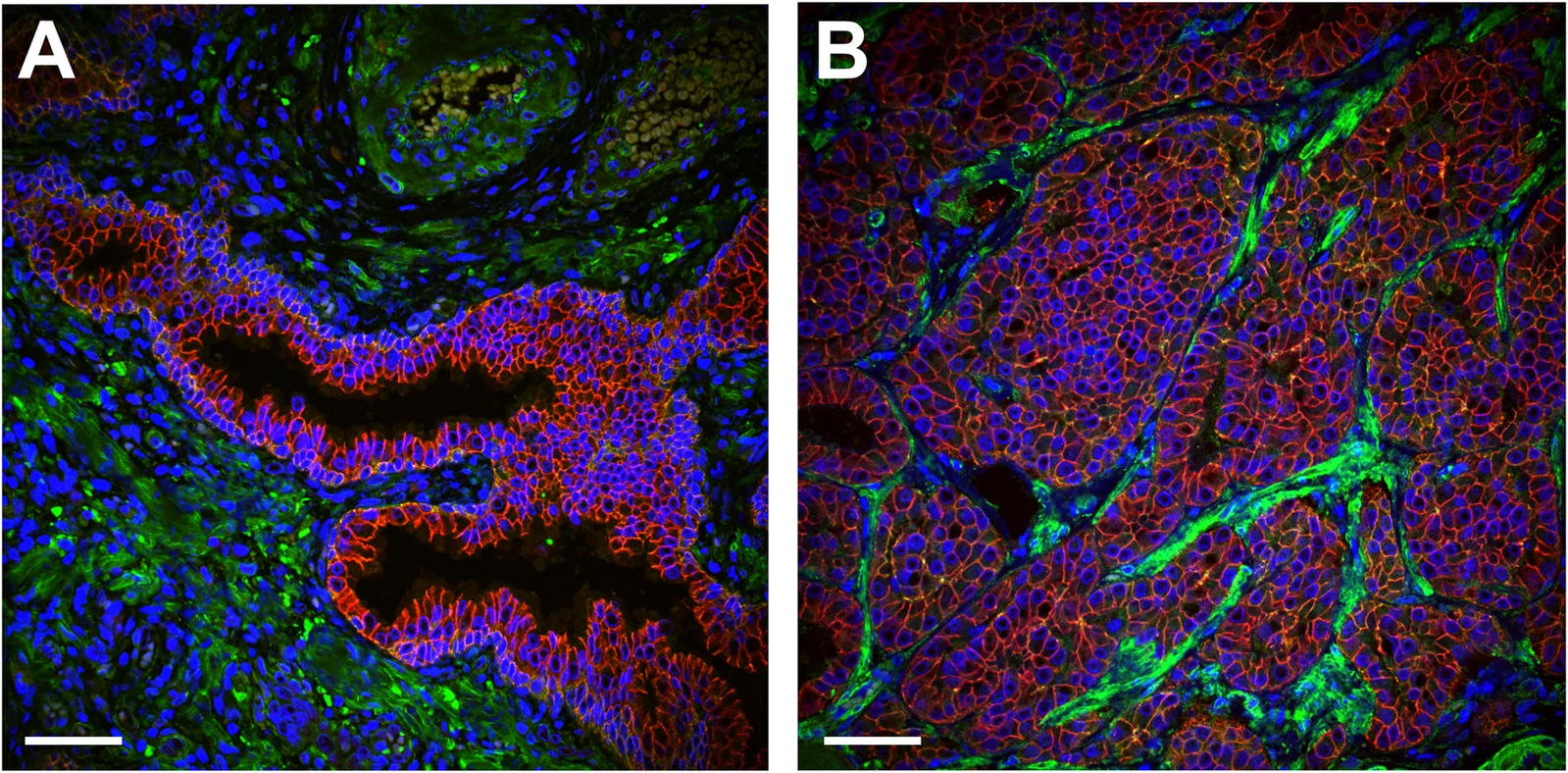
Immunofluorescence image of tissue used to derive UCSF-PR2. E-cadherin (cell-cell adhesion marker, red), vinculin (focal adhesion marker, green), DAPI (nuclear marker, blue). **(A)** Benign prostatic tissue (used for organoid UCSF-PR2). **(B)** High-grade prostate tumor (Gleason Score 5 + 4), observe higher expression of E-cadherin. Scale bars equal 30 µm.

Next, we made an effort to optimize the organoid medium composition to select for prostate tumor cells. Because normal and tumor tissue have distinctly different requirements for matrix-dependent signaling (see Figure 4, an increased E-cadherin expression in tumor tissue compared to the benign tissue used for organoid UCSF-PR2), correct modulation of contractility is important for successful tumor organoid establishment. Y-27632 is an inhibitor of Rho kinase (ROCK), which inhibits myosin light chain phosphorylation and as a result myosin activity (Matov, 2024c; Matov, 2024h; Matov, 2025b; Narumiya et al., 2000). The inhibition of acto-myosin contractility is advantageous for establishing 3D organoid cultures (Sachs and Clevers, 2014) because it modulates the mechanical interactions with the extracellular matrix provided by the Matrigel. Y-27632 is added during phases of establishment of prostate organoids from single cells (Drost et al., 2016) to prevent apoptosis during the procedure, which includes long-term suspension and soft Matrigel culture. By seeding organoids in different wells with different levels of the drug in the medium, we observed that cells from benign tissues are considerably more dependent on Y-27632 (see the effects of 70 µM of the myosin II inhibitor blebbistatin – which accurately replicates all of the effects of Y-27632 – on the contractility of the actin cytoskeleton in (Matov, 2024h)) ROCK inhibitor to form organoids. Also, we have found empirically that cells derived from high-grade prostate tumors are resilient (Green, 2024) to apoptosis and have consequently been able to temporarily select tumor cells by reducing the concentration of Y-27632 ROCK inhibitor 4-fold (to 2.5 µM) in comparison to the protocol (Drost et al., 2016) for normal prostate tissue organoids.

It is conceivable that PC that is different histologically would require a different concentration of Y-27632 ROCK inhibitor, which could also provide clues for therapeutic approaches. Low-grade PC tumors (Gleason Score 3) convert to normal epithelium when cultured *ex vivo* as organoid cells (data not shown) or tissue (personal communications). As much as such results seem unlikely, they might indicate that early-stage PC might be a purely histological phenomenon which reverses when the hypoxia-inducible factor 1 α (HIF1α) and the glycogen synthase kinase 3 (GSK3) are downregulated; they also suggest a strategy to reverse the transformations occurring in an aging prostate. Distinctly, GSK3α is elevated in low Gleason Score tumors, while GSK3β is overexpressed in high Gleason Score tumors (Duda et al., 2020) and the isoform-specific differentiation suggests different treatment approaches. GSK3 has been demonstrated as a positive regulator in AR transactivation and PC growth independent of the Wnt/β-catenin pathway. Different types of GSK3 inhibitors including lithium show promising results in suppressing tumor growth in different animal models of PC (Li et al., 2015). High-grade PC tumors (Gleason Score 4, 5) do not propagate *ex vivo* as most terminally differentiated primary tumors are dormant (Bill-Axelson et al., 2018). Terminally differentiated primary tumor cells divide once or twice per year (Thadani-Mulero et al., 2014) and are quickly overgrown by normal prostate epithelial cells, as the organoid medium is tailored for culturing of normal cells. In this context, identifying the optimal medium composition for culturing of primary PC organoids will effectively equate to providing a treatment strategy for localized disease.

#### Androgen-independent PC

Although early-stage PC can be treated reasonably well clinically, advanced forms of this disease are very aggressive and difficult to manage with existing therapies. Consequently, metastatic tumors are associated with a high degree of morbidity and mortality. In the metastatic setting, assays to detect CTCs in the peripheral blood of cancer patients have been used clinically to provide predictive and prognostic information (Sung et al., 2013; Sung et al., 2012; Tasaki et al., 2014). Even if the use of microfluidic devices to isolate CTCs from peripheral blood for diagnostics and disease progression prognosis has gained popularity (Cristofanilli et al., 2004; Yu et al., 2011), such approaches carry the risk of missing CTCs during the cell capture stage. An alternative is to utilize platforms that comprise imaging all cells, without enrichment, similar to the “no cell left behind” approach (Cho, 2012), which had a slogan borrowed from the American administration regarding education at the time of conceiving the method. Surprisingly, the approach relied on large size CTCs only, the so-called HD (standing for “high definition”) CTCs, which were considered to be the detections of a high level of certainty, and disregarded, for example, cells with diameter similar to that of leukocytes, thus generating a sizable number of false negative selections. The reason behind this size determination was the assumption that the tumor cells present in circulation are larger than normal prostate or other epithelial cells detected in circulation and 2-fold larger than leukocytes (Cho, 2012). While this assumption might hold true for many (but certainly not all) of the CTCs while the disease still responds to ADT, it is unlikely to be true once the tumors are castrate-resistant and the CTCs (Vid. 1) become androgen independent.

**FIGURE 5:**
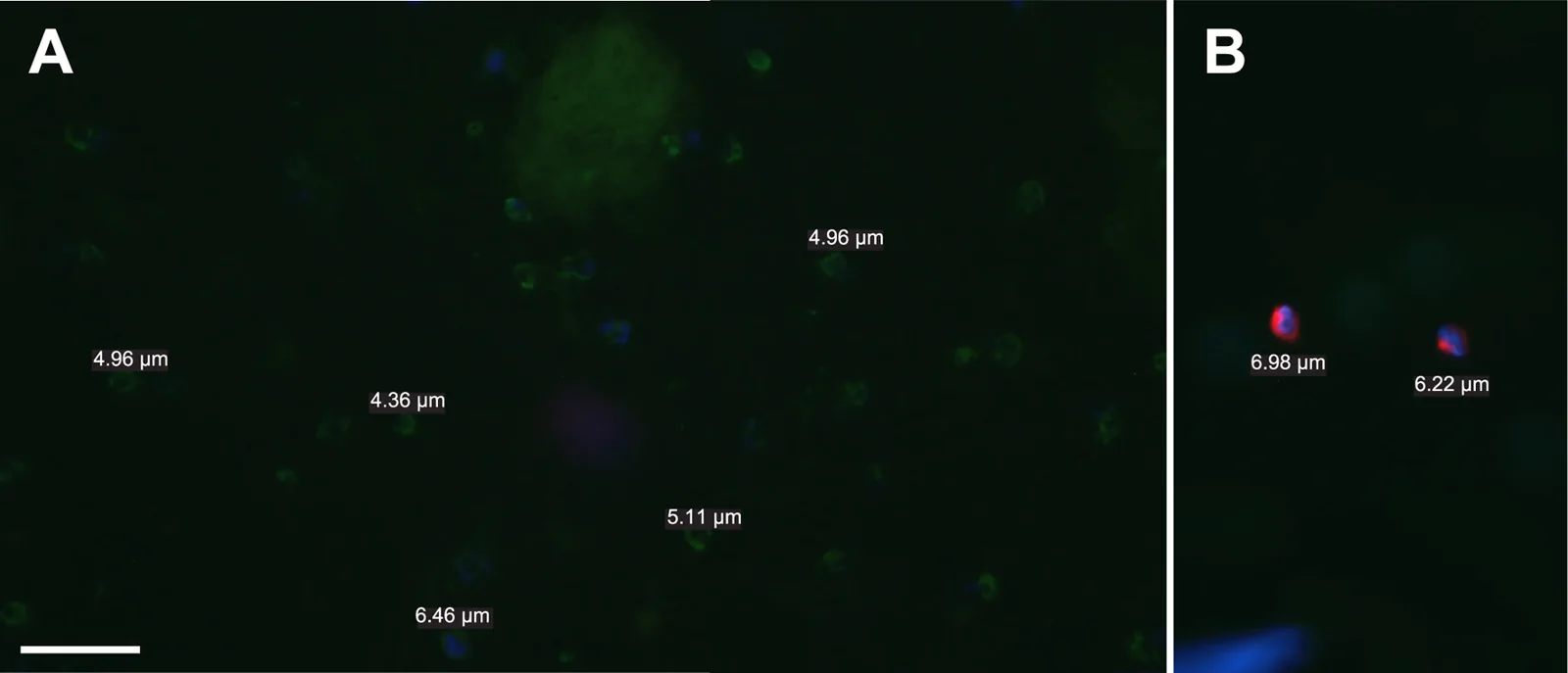
CTCs with diameter smaller than that of a normal prostate epithelial cell appear when the disease progresses to become androgen independent (or mCRPC). Figure legend: PSMA (J591, tumor marker, red), CD45 (leukocyte marker, green), DAPI (nuclear marker, blue). The scale bar equals 20 µm. **(A)** The diameter of patient leukocytes is between 4.3 µm and 5.2 µm. **(B)** The diameter of patient CTCs is as small as 6.2 µm-7.0 µm after the disease becomes castrate-resistant.

To avoid the effects of therapy and acquire resistance to androgen inhibitors, prostate tumors bypass the need for androgen (Bander et al., 2005), which affects their metabolic and cell growth patterns. While undetectable via AR expression, such CTCs can be detected by the expression of prostate-specific membrane antigen (PSMA), a cell surface marker present and enriched in 75%–95% of mCRPC tumors (Vlachostergios et al., 2021). Such androgen-independent tumor cells have differential regulation and are much smaller in size (Figure 5). While leukocytes are around 5.2 µm in diameter (Figure 5A), the CTCs of a mCRPC patient can be about just 27% larger or around 6.6 µm in diameter (Figure 5B). These results, albeit not statistically representative because of their rare event nature, indicate that in a peripheral blood draw collected from a mCRPC patient, large CTCs (of 7-13 µm in diameter or larger (Supplementary Figure S2)) could be completely absent, yet the disseminated disease might be present through a very few significantly smaller (<7 µm in diameter) tumor cells. These small CTCs can be identified via quantitative microscopy methods (Matov, 2024g) and eliminated through PSMA-targeted therapy (Unterrainer et al., 2024). In addition, mCRPC tumors often bypass the functional requirement for AR through epigenetic cellular plasticity, evidenced by the manifestation of neuroendocrine transdifferentiation (Davies et al., 2018). FGF signaling can bypass a requirement for AR, and FGF and MAPK pathways are active in metastatic AR-null PC (Bluemn et al., 2017). MAPK targeting might be efficacious in inducing ferroptosis (Wang et al., 2023) in prostate tumors with an AR-null phenotype for *TP53*-altered patients, for whom the time to an endpoint of no longer clinically benefitting from AR pathway inhibitors and taxanes is comparable (De Laere et al., 2024).

Although not frequently mutated in PC, RAS isoforms play a pivotal role in multiple pathways that have been implicated in PC progression to androgen independence, including growth factor and cytokine-induced activation of AR and its co-regulators by post-translational modifications (Whitaker and Neal, 2010). Both tubulin and actin subunits polymerize head-to-tail, thus creating polar filaments. Cytoskeletal protein complexes can be several dozen micrometers long and reach opposite sides of a cell. However, the molecular subunits of the filaments are in the nanometer scale range. For this reason, cellular structures can undergo rapid structural reorganizations by depolymerizing filaments at one site while polymerizing elsewhere in the cell. Actin filaments are polymers of actin monomers, and MTs consist of α/β tubulin dimers. The non-equilibrium intracellular dynamics of both filamentous actin (F-actin) and MTs are largely determined by energy derived from ATP or GTP hydrolysis and polymerizing MTs consist of GTP-bound tubulin dimers at their growing tips; hydrolysis follows, and the MT lattice consists of GDP-bound tubulin. RAS is activated by GTP loading and deactivated upon GTP hydrolysis to GDP. Therefore, healthy MTs would compete with oncogenic RAS for GTP, and one could indirectly limit RAS’ activity by restoring the normal functioning of the MT cytoskeleton (Basseville et al., 2015), which is dysregulated in cancer (Ertych et al., 2014; Galletti et al., 2014; Gonçalves et al., 2001; Harkcom et al., 2014; Kuo et al., 2025; Thoma et al., 2010) and regulated by functional pVHL (Thoma et al., 2010). RAS-driven cancers have proven very difficult to treat (Hong et al., 2020; Stephen et al., 2014; Zhang et al., 2018) and the most common *KRAS* p.G12C mutation can be targeted via proteins fused to the pVHL E3 ubiquitin ligase (Bery et al., 2020; Bond et al., 2020). Using proteolysis targeting chimeras to degrade proteins linked to tumorigenesis has emerged as a potential therapeutic strategy for cancer (Khan et al., 2020). These heterobifunctional molecules consist of one ligand for binding to a protein of interest and another to an E3 ligase, connected via a linker, and only a few E3 ligases can be used to degrade target proteins with this approach, including pVHL (Zhao et al., 2019). In PC, proteolysis targeting chimera agents that utilize pVHL E3 ligase ligands (Han et al., 2019) represent promising AR-targeting therapeutics and could become an important part of disease treatment (Zang et al., 2024) as oral protein degradation agents have shown encouraging results in clinical trials (Qi et al., 2021).

Op18, a MT polymerization inhibitor (Cassimeris, 2002), has been associated with taxane resistance in androgen-independent PC (Mistry and Atweh, 2006). In meiotic spindles, we discovered that the upregulation of Op18 leads to the formation of about 3-fold smaller spindles (Houghtaling et al., 2009) and it inhibited the formation of a particular subset of MT bundles that directly link the two spindle poles (termed non-kinetochore MTs). The non-kinetochore MTs represent a distinct MT subpopulation and polymerize at slightly higher rates (about 0.3 μm/min faster) than those MT bundles that attach to the kinetochores, which have their dynamics tightly regulated by the protein complexes at the kinetochore (Matov, 2025b; Vallotton et al., 2003). We measured a dose-dependent decrease in spindle size upon addition of up to 2.5-fold excess Op18 (endogenous Op18 concentration of 6 μM (Belmont and Mitchison, 1996), 15 μM of recombinant Op18 added) without significantly reducing MT polymerization rates (<1 μm/min attenuation) (Houghtaling et al., 2009; Matov, 2025a). In interphase cells, our analysis of gene expression data of four PC patient organoids refractory to androgen therapy (Supplementary Figure S3) indicated upregulation of Op18 in the patient organoid for which we measured lower MT polymerization rates (>3 μm/min attenuation) (Matov, 2024e). Taken together, the data indicate that in androgen-independent PC tumors the upregulation of Op18 might induce a reduction of cell size and resilience to therapeutic interventions. Overall, our work has identified differential mechanisms of cytoskeletal remodeling in epithelial tumors leading to either very large spindles or very small spindles in cells that are drug resistant.

#### Small molecules’ effects on MT dynamics

An important process for tumor development and metastasis involves the remodeling of the 3D microenvironment surrounding the tumor cells. Dynamic remodeling of the tumor microenvironment is a critical prerequisite step for metastatic tumor growth driven by signaling between cancer cells and cells in the surrounding extracellular matrix. MT cytoskeleton reorganization regulates the signaling processes that promote the remodeling of the tumor microenvironment and drug resistance. For example, the fusion of *TMPRSS2* with *ETS* genes is a recurrent genomic alteration in PC and the ETS-related gene (*ERG*) is the most common fusion partner for the androgen-regulated gene *TMPRSS2* (Tomlins et al., 2005). ERG expression affects several parameters of MT dynamics and inhibits effective drug-target engagement of docetaxel or cabazitaxel with tubulin (Matov et al., 2013). In CTCs captured from an ERG-negative patient, we observed robust drug-target engagement in 41 of the 66 captured CTCs (62%) and in 82 of the 105 captured CTCs (78%) following docetaxel and cabazitaxel treatment, respectively (Galletti et al., 2014). In contrast, in the CTCs isolated from an ERG-positive patient, there was no increase in MT bundling with either treatment and MT bundle formation is the result of drug-mediated MT polymer stabilization, and as such, it represents a hallmark of taxane cellular activity (Tasaki et al., 2014).

**FIGURE 6:**
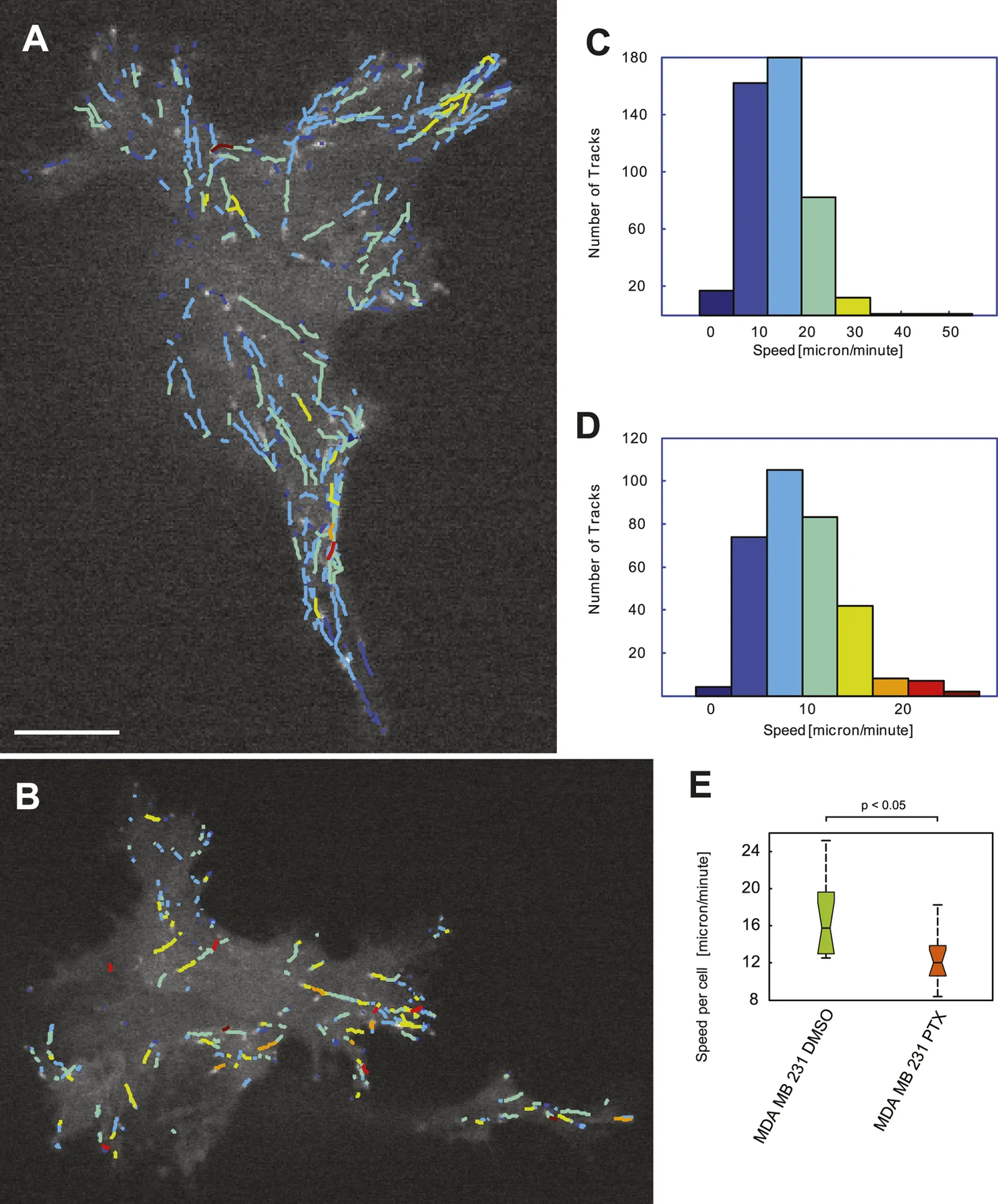
Paclitaxel attenuates MT growth rates in breast cancer (MDA-MB-231) cells. MDA-MB-231 cells were transiently transfected with EB1ΔC-2xEGFP and treated with vehicle (DMSO) or 100 nM paclitaxel for 2 h. EB1ΔC-2xEGFP comet time-lapse sequences were recorded every 0.5s for 125 frames and computationally tracked (Yang et al., 2005). The color-coding represents EB1 comet speeds; colder colors correspond to lower speeds, and warmer colors correspond to faster speeds. The scale bar equals 10 µm. An overlay of EB1 comets raw data and computer-generated growth tracks with a minimum lifetime of four frames (or 1.5s) is shown in **(A)** for DMSO, for which the median speeds were about 11 μm/min and the maximal speeds were about 49 μm/min and in **(B)** for paclitaxel, for which the median speeds were about 8 μm/min and the maximal speeds were about 25 μm/min. Histograms of EB1 comet speeds in µm/min are shown in **(C)** for DMSO and **(D)** for paclitaxel. **(E)** Distributions of the speed of 155,981 EB1 comets in 14 DMSO-treated (left) and 39,989 EB1 comets in 14 paclitaxel (PTX) treated cells (right), and of per-cell mean speed. The significance of the difference between concentrations was determined by a permutation *t*-test (p < 0.05).

Next, to evaluate the effects of metastatic, receptor-negative epithelial tumors on the MT cytoskeleton, we measured MT polymerization dynamics using automated motion tracking of EB1/3ΔC-2xEGFP comets (Harkcom et al., 2014; Kumar et al., 2009; Matov et al., 2016; Matov et al., 2013; Matov et al., 2005; Thoma et al., 2010). We have shown that there exists a rocking pendulum-like motion in many of the mitotic spindles in MDA-MB-231 cells (Matov, 2024e). The MDA-MB-231 cells have the largest spindles and, while most of the spindles in other cell lines remain stationary until anaphase, many of them rotate at up to an angle of ±40° (Matov et al., 2015) to the left and right of the polar spindle axis. This rapid, rocking spindle motion over an 80° angle is reflected in the increased EB1 comet speeds during interphase. Altered MT dynamics endow cancer cells with both survival and migratory advantages (Mitchison, 2012), which can be correlated with drug resistance (Gonçalves et al., 2001). In interphase cells, our results show that a 2-h treatment with 100 nM paclitaxel brings MT dynamics to a very uniform behavior (Tran et al., 2013; Tran et al., 2012) by suppressing the fast polymerizing MTs and lowers significantly the overall polymerization rates (Figure 6). Tracking of EB1 comets in a MCF10A normal breast epithelium cell line indicated that the EB1 comets in cells from normal breast epithelium move about 30%-70% faster in comparison to breast cancer cells (see Supplementary Figure S4 and (Matov, 2024e)).

Besides differential regulation in disease, the size of the cells and the mitotic spindles they form also affect MT dynamics. We have shown that higher EB1 density decreases MT growth rates (Geisterfer et al., 2020; Jevtic et al., 2014; Jevtic et al., 2013), which can lead to taxane resistance as well (Galletti et al., 2014; Matov, 2024e). Taken together, these data suggest that smaller tumor cells are also more likely to be resistant to therapy.

Nicotinamide adenine dinucleotide (NAD^+^) is an endogenous small molecule that has effects on diverse processes, including obesity, lifespan, and cancer, which can increase MT polymerization rates (Harkcom et al., 2014) and restore normal MT regulation and homeostasis, which suggests its utilization during the treatment of tumors of epithelial origin. However, there have thus far been no studies on the effects of the essential redox metabolite NAD^+^ specifically in PC. NADH elevation during chronic hypoxia leads to pVHL-mediated HIF1α degradation (Joo et al., 2023), which has metabolic consequences for quiescent cells. HIF1α drives resistance to ferroptosis in solid tumors (Yang et al., 2023) and a transient increase of HIF1α during the G1 phase prevents the onset of starvation-induced apoptosis (Belapurkar et al., 2023). GSK3β also plays a role in modulating ferroptotic sensitivity by regulating cellular iron metabolism (Wang et al., 2021). In bacterial antibiotic resistance, a non-mutational mechanism of acquired drug resistance is the state of persistence (Fisher et al., 2017) during which a small population (<5%) of the cells become quiescent. In cancer, the quiescent surviving persister cells minimize the rates of proliferation and, thus, become not susceptible to treatment with mitotic inhibitors and targeted therapies (He et al., 2024; Russo et al., 2024). This reversible process of becoming drug-tolerant (Green, 2024) is linked to the upregulation of mesenchymal markers and downregulation of epithelial markers in the persister cells. During drug holiday, the cells revert to normal metabolism and expand their population, while becoming again susceptible to drug treatment. This cycle repeats multiple times during patient treatment. While the tumor is under drug pressure, however, the few drug-tolerant persister cells pay a metabolic price and become temporarily vulnerable to GPX4 inhibition (Hangauer et al., 2017). We have investigated the effects of small molecules, RSL3 and ML210, which inhibit the activity of GPX4 and induce ferroptosis in, initially drug-sensitive, patient cells in PC organoids with non-mutational acquired drug resistance (Matov, 2024b) and identified a clinical approach to impede disease relapse and eliminate residual disease (Hangauer et al., 2017). Ferroptosis has a dual role in cancer. It plays a role in tumor initiation and tumorigenesis, which depends on inflammation-associated immunosuppression triggered by ferroptotic damage (Chen et al., 2021) and later, during treatment, in tumor suppression (Hangauer et al., 2017). Recently, we identified the presence of patterns of DNA fragmentation linked to a decrease in the number of caspase-dependent DNA cleavage fragments with lengths 198 bp, 364 bp, and 521 bp, and differential detoxification of reactive oxygen species and metabolic/redox patterns in colorectal cancer (Matov, 2024a). NAD^+^ promotes cellular oxidative metabolic reactions and enables mitochondrion respiration to support cell growth and survival (Griffiths et al., 2020). Taken together, these results suggest the utilization of NAD^+^ and other endogenous molecules in the treatment of PC in the context of modulating the drug susceptibility of resistant PC, that is, as drug regimen sensitizing compounds.

#### GSK3β and pVHL maintain MT dynamicity

Mutations in the *VHL* gene lead to various patterns of MT dynamics signatures (Thoma et al., 2010). pVHL regulates the activity of HIF1α (Genbacev et al., 2001; Kaelin and Ratcliffe, 2008; Maxwell et al., 2001; Staller et al., 2003) and *VHL* mutations lead to a variety of malignant and benign tumors of the eye, brain, spinal cord, kidney, pancreas, and adrenal glands. The hypoxic microenvironment drives the selection of aggressive cancer cells through actin cytoskeleton polarization, downregulation of E-cadherin, and epithelial-mesenchymal transition (Buart et al., 2021). Mutations in *VHL* lead to an increased GTP hydrolysis (Thoma et al., 2010) and dysregulated MT dynamics (Barry and Krek, 2004), a decreased drug susceptibility, and increased oncogenic activity. Dysfunctional pVHL tumor suppressor leads, for instance, to the upregulation of the MT-associated protein MAP1, which has been implicated in pathogenesis (Czaniecki et al., 2019; Fifre et al., 2006) as well as drug resistance in several cancers (Laks et al., 2018) and, thus, represents a target within the MT transcriptome to sensitize resistant disease. We have demonstrated that it is possible to associate dysregulation of specific aspects of MT dynamics to mutations occurring within a particular domain of pVHL (Thoma et al., 2010). Some tubulin inhibitors bind to individual tubulin dimers and block tubulin polymerization (MT-destabilizing drugs); others bind on the lumenal side of the MT lattice and inhibit MTs’ ability to depolymerize (MT-stabilizing drugs). Mutations in the MT-interacting/HIF1α-interacting domain are more often linked to changes in MT polymerization/depolymerization (or turnover) rates (for example, Y98H mutation), and therefore, such disease could be treated with MT-destabilizing drugs. *VHL* mutations in the elongin C-interacting domain are more often linked to changes in the MT catastrophe and rescue frequencies (for example, R161P mutation), and therefore, such disease could be treated with MT-stabilizing drugs.

Signaling through HIF1α is dependent on interphase MTs, and the protein accumulation and translational regulation of HIF1α is inhibited by taxane treatment (Carbonaro et al., 2012; Carbonaro et al., 2011). Activation of quiescent fibroblasts by tumor cells is dependent on the integrity of the tumor cell MT cytoskeleton and, thus, taxane treatment reduces fibroblast activation as a result of drug-mediated suppression of MT dynamics (Tran et al., 2013; Tran et al., 2012). We also identified an isoform-specific attenuation of MT polymerization rates in *VHL* ^−/−^ renal cell carcinoma cells in which either the short or long pVHL isoforms were reconstituted. Viral reconstitution of pVHL19 partially reduced the EB3 comet speeds (with 2.1 μm/min) and increased the times of MT pause by 1s on average, while pVHL30 further reduced the EB3 speeds (with 4.1 μm/min) but increased the times of MT pause by just half a second on average (Thoma et al., 2010). Therefore, the overall attenuation of MT polymerization rates for both pVHL isoforms was very similar, but demonstrated differential isoform-specific mechanisms, which may represent a personalized targeting approach. In addition, we identified the mechanism by which pVHL30 protects MTs from depolymerization after treatment with 40 nM nocodazole; while MT tips in *VHL* ^−/−^ renal cell carcinoma cells instantly get sparser upon adding the drug (Vid. 2) and within 6 min depolymerized when co-cultured with the drug, in cells with reconstituted pVHL30, MTs paused significantly longer and thus resisted the drug treatment.

**FIGURE 7:**
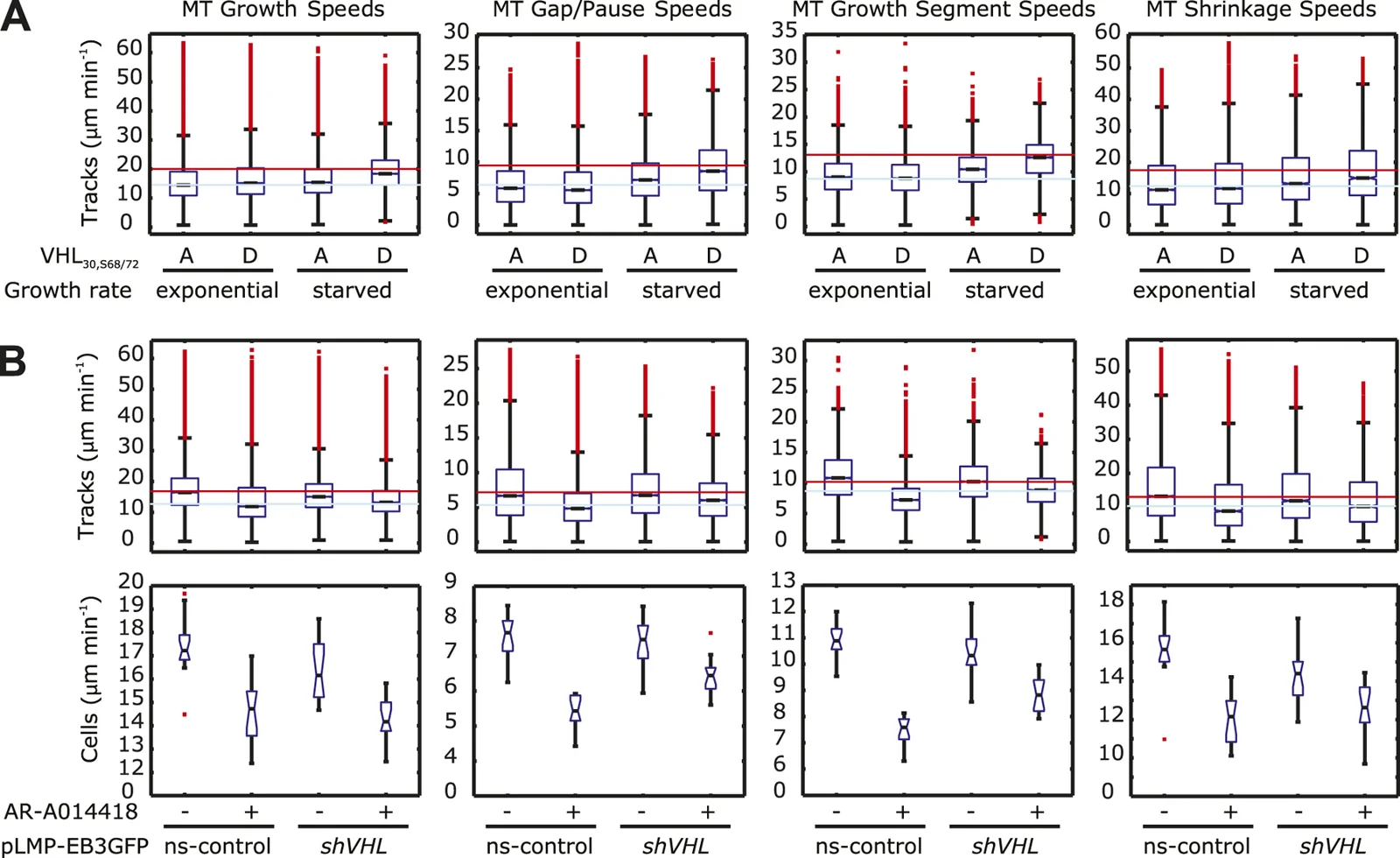
pVHL and GSK3 affect MT dynamics in interphase and quiescent cells. MT tips are labeled with EB3ΔC-2xEGFP, imaged for a minute (with an acquisition rate of two images per second) and computationally analyzed (Matov et al., 2010). About a dozen cells were analyzed per condition, containing about 4,000 MT tracks each with a minimum track lifetime of 1.5s. The four MT dynamics parameters (Matov et al., 2010) compared are as follows: (1) MT polymerization rates (growth speeds), (2) MT dynamics during trajectory gaps, when the EB3ΔC-2xEGFP molecular marker is not visible (pause/gap/flux speeds), (3) MT polymerization rates over multiple trajectories interrupted by periods when the EB3ΔC-2xEGFP molecular marker is not visible (growth segment speeds), (4) MT depolymerization rates (shrinkage/shortening speeds). **(A)** Box plots representing the measured MT-dynamics parameters in RCC-4 cells reconstituted with the phosphorylation site mutant pVHL30(S68/72A) resistant to phosphorylation by GSK3β and the phosphorylation-mimicking mutant pVHL30(S68/72D) under exponential growth and serum-starved conditions. For comparison, each parameter’s median value is indicated for RCC-4 cells reconstituted with pVHL30 under exponential growth (light blue line) and serum-starved (thin red line) conditions. **(B)** Box plots representing the measured MT-dynamic parameters per track (upper panels) and per cell (lower panels) of a non-silencing control (ns-control) RPE-1 cells and cells depleted of pVHL using retroviral expression of microRNA-based small hairpin RNA (shRNAmir) (via cloning into pLMP-EB3GFP retrovirus vector (Hergovich et al., 2006)) against *VHL* (*shVHL*) under serum-starved conditions. To get the cells in a quiescent state, they were grown for 24 h and then serum-starved for 24 h. For GSK3 inhibition, 20 h of starvation was followed by incubation with 100 μM AR-A014418 for 4 h in the same medium. For comparison, each parameter’s median value for MT tracks is indicated for a non-silencing control (ns-control) RPE-1 cells (light blue line) and cells depleted of pVHL using retroviral expression of microRNA-based small hairpin RNA under exponential growth conditions (thin red line). Per-cell measurements are added because the cell-to-cell variation is the single biggest source of variation in the median value of a MT parameter. The decrease in the values for each parameter after the addition of AR-A014418 was statistically significant.

Further, analysis of pVHL species carrying non-phosphorylatable or phospho-mimicking mutations at Ser-68 and Ser-72 revealed a central role for phosphorylation events in the regulation of pVHL’s MT stabilization (Hergovich et al., 2006). Our measurements (Table 1) of MT dynamics showed that in cells stably transfected with retroviral vectors expressing the pVHL-GSK3β phosphorylation-site mutants (S68/72A and S68/72D) different alterations in MT homeostasis have been induced (Figure 7). pVHL mutant S68/S72A (linked to cilium rescue when GSK3β is subjected to inhibitory phosphorylation (Thoma et al., 2007a)), which is resistant to phosphorylation by GSK3β, leads to reduced MT growth dynamics of 15.8 μm/min (Table 1) and increased MT pausing after 40 nM nocodazole treatment, similar to the effects of reconstituting the long pVHL isoform. GSK3β and pVHL cooperatively maintain primary cilia (Davenport and Yoder, 2005) in RCC-4 cells; pVHL plays a key role in maintaining the structural integrity of the primary cilium, a MT-based cellular antenna important for suppression of uncontrolled proliferation of kidney epithelial cells and cyst formation. The cilium maintenance function of pVHL is independent of its role in HIF1α degradation, but it is dependent on its ability to stabilize the MT network of the ciliary axoneme (Thoma et al., 2007a). pVHL-negative cells lose their primary cilia in response to serum stimulation. The effect of serum on pVHL-negative cells is dependent on PI3K signaling and could be mimicked by inhibition of GSK3β. Thus, pVHL and GSK3β demonstrate a genetic redundancy with respect to cilia maintenance; inhibition or loss of either protein alone has no effect on the primary cilium, but inhibition or loss of both causes cells to rapidly lose their cilia (Thoma et al., 2007b). Overall, our analysis shows that growth factor deprivation enhances MT turnover and destabilizes MT, and that GSK3β-mediated phosphorylation and inhibition of pVHL function induce these changes in MT homeostasis.

**TABLE 1:** Loss of pVHL leads to MT turnover rates (in red) similar to quiescent cells with phosphorylated pVHL.

Cells	Mutation	Type	MT tip speed	Videos	MT tracks	Cilia
RCC-4	Null	Loss of pVHL	18.7 μm/min	10	37,120	No
RCC-4	None	Long isoform	14.8 μm/min	9	41,844	Yes
RCC-4	None	Short isoform	16.8 μm/min	12	28,578	Yes
RCC-4	S68/72A	GSK3 non-phosphorylatable	15.8 μm/min	30	166,669	Yes
RCC-4	S68/72D	GSK3 phosphorylation	16.7 μm/min	28	102,202	No
RPE-1	ns	Long isoform	15.4 μm/min	12	68,939	Yes
RPE-1	shVHL	Loss of pVHL	17.6 μm/min	13	55,286	No
RPE-1	ns s-starved	Long isoform 100 μM DMSO	17.6 μm/min	15	39,541	N/A
RPE-1	ns s-starved	100 μM GSK3 inhibitor VIII	14.6 μm/min	13	35,515	N/A
RCC-4	s-starved	Long isoform	*20.3 μm/min*	*6*	*8,590*	N/A
RCC-4	S68/72A s-starved	GSK3 non-phosphorylatable	16.7 μm/min	13	34,778	N/A
RCC-4	S68/72D s-starved	GSK3 phosphorylation	19.3 μm/min	12	22,222	N/A

Conversely, in cells with reconstituted pVHL, MTs dynamics (in blue) are similar to quiescent cells with inhibited GSK3. Computational analysis of MT dynamics was performed using ClusterTrack (Matov et al., 2010). The cell lines consist of clear cell renal cell carcinoma (ccRCC) cells and hTERT-immortalized retinal pigment epithelial (RPE-1) cells (Vid. 3). RCC-4 cells, which are deficient for pVHL, were engineered to stably produce the empty vector as control (pCMV(R)-VHL^−/−^), the long or short pVHL isoforms (pCMV(R)-VHL30 and pCMV(R)-VHL19, respectively), and the phosphorylation site mutant pVHL30(S68/72A), which is resistant to phosphorylation by GSK3β or the phosphorylation-mimicking mutant pVHL30(S68/72D). RPE-1 cells, which express wild-type pVHL, were transfected with retroviral vectors expressing a non-silencing control (ns-control) or a miRNA30-based short-hairpin RNA to knockdown pVHL (shVHL) (Vid. 4). To enter a quiescent state, cells were serum-starved for 24 h. The GSK3 inhibitor used was VIII (AR-A014418) at concentration of 100 μM. For the 12 conditions, a combined number of 173 movies containing more than 640,000 MT tracks with duration of at least 1.5s were analyzed. Each movie consists of 125 image frames taken 0.5s apart. Cilia maintenance data is incomplete for five of the conditions (serum-starved cells). Conditions for which the MT polymerization rates are different with statistical significance (p < 0.05) have the EB1 comet speed numbers shown in red (higher rates) or blue (lower rates). The 10^th^ condition (RCC-4 serum-starved cells expressing the long isoform of pVHL) is based on only six videos (with 1 or 2 cells) and fewer MT tracks than normally detected per video (datasets of at least either 10 videos or 20,000 MT tracks are considered), and some of the cells were either much smaller than in any other condition or represented an unusual morphological outlier; these factors are indications of a statistically not representative dataset and poor data quality and, thus, the analysis result (in italic) constitutes an artifact.

#### MT regulation in quiescent cancer cells

Phosphorylation of pVHL at Ser-68 proceeds mainly in serum-starved cells and is consistent with the fact that GSK3β is predominantly active under conditions of serum starvation (Lochhead et al., 2006). Our measurements of EB3 comet speeds after perturbations of the GSK3β-pVHL axis indicated altered MT dynamic instability. Specifically, serum-starvation of pVHL-positive RPE-1 cells altered MT turnover to the same extent as shRNA-mediated depletion of pVHL in exponentially-growing cells when serum was present (Figure 7; Table 1). Importantly, inhibition of GSK3β in serum-deprived cells attenuated MT turnover, which reached rates similar to those we measured in exponentially-growing, pVHL-positive RPE-1 cells. To achieve GSK3 inhibition, 20-h starvation was followed by a 4-h incubation with 100 μM GSK3 inhibitor VIII (AR-A014418), which is used in the treatment of PC (Li et al., 2015). Our results suggest that the state of quiescence is characterized by increased MT dynamics, as compared to the state of exponential growth, and that GSK3β plays a critical role in mediating the changes in MT dynamics (Table 1). To assess whether GSK3β-mediated phosphorylation of pVHL contributes to the measured changes in MT dynamics associated with quiescence, we analyzed serum-starved RCC-4 cells producing either the phosphorylation site mutant pVHL30(S68/72A) that is resistant to phosphorylation by GSK3β or the phosphorylation-mimicking mutant pVHL30(S68/72D) (Figure 7; Table 1). Serum deprivation caused an increase in MT turnover in RCC-4 cells that expressed pVHL30 or pVHL30(S68/72D) but not in cells expressing pVHL30(S68/72A). Changes in MT dynamics such as an increase in MT turnover upon serum deprivation were coupled with a decrease in MT shrinkage times, that is, an increase in MT catastrophe frequencies. Our results suggest that phosphorylation of pVHL at Ser-68 by GSK3β inactivates the MT stabilization functions of pVHL in terms of attenuation of MT turnover and inhibition of catastrophe events, and indicate that phosphorylation is a critical aspect of the regulation of MT dynamics when cells enter a quiescent state.

#### MTs in metastatic cells

The cytoplasmic linker protein CLIP-170, by recognizing conformational changes, like EB1/3, binds growing MT tips and forms comets (Vid. 5), and has been linked to taxane resistance (Thakkar et al., 2021). In migrating epithelial cells, such as cells undergoing epithelial-mesenchymal transition, cytoplasmic linker-associated proteins (CLASPs) bind (or track) selectively MT growing tips in the cell body, but bind along the MT lattice in the lamella. GSK3β phosphorylates cytoplasmic CLASPs at multiple sites in the domain required for MT growing tip interaction. Expression of constitutively active GSK3β destabilizes lamella MTs by disrupting lateral MT interactions with the cell cortex.

GSK3β-induced lamella MT destabilization (Kumar et al., 2009) can be partially rescued by the expression of CLASP2 with mutated phosphorylation sites indicating that CLASP-mediated stabilization of peripheral MTs, which likely occurs in the vicinity of focal adhesions (FAs), is regulated by local GSK3β inactivation. Similar to the effects of functional pVHL (Thoma et al., 2010), ectopic activation of GSK3β at the cell edge results in the inhibition of MT catastrophe events (Kumar et al., 2009). When MTs switch to a state of depolymerization less often, cells become larger, the MTs get longer and remain as polymers for an extended period of time – and that leads to MT acetylation (Janke and Montagnac, 2017), which also (further) inhibits MT catastrophe events (Matov et al., 2010). MT post-translational modifications in α-tubulin that induce an increase in polymer rigidity and mechanical resistance, such as detorysination and acetylation, stabilize MTs (Eshun-Wilson et al., 2019) and also induce drug resistance (Galmarini et al., 2003; Kavallaris, 2010; Matov et al., 2010). Overall, our investigation indicates that both faster than normal MT turnover rates and slower than normal MT turnover rates are hallmarks of drug resistance.

### Anticipating drug resistance

#### Resistance evaluation via circular Hough transform

**FIGURE 8:**
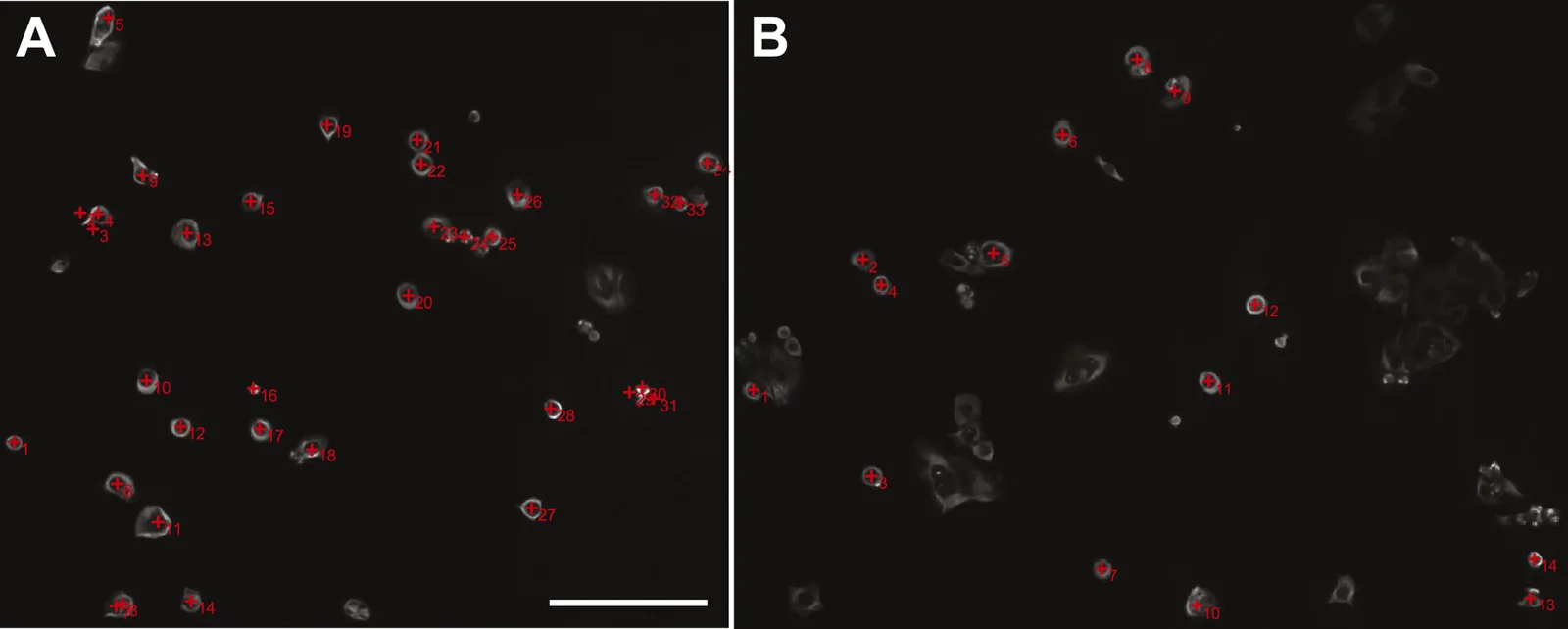
Paclitaxel resistance resulting from knockdown of BRCA1 in adenocarcinoma (A549) cells. Cells were treated with 100 nM paclitaxel for 18 h. Cell center in drug-affected cells is marked with a red +. Cells with bright intensity rings in α-tubulin immunofluorescence images (Sung and Giannakakou, 2014) are detected and enumerated. Scale bar equals 100 µm. **(A)** A549 cells respond to paclitaxel treatment. In this field of view, 34 cells responded (83%) to the treatment and their MT cytoskeleton formed round bundles, 4 cells were not affected (9.7%) by the drug and did not form bundles, and 3 cells were already apoptotic (7.3%) by the time of imaging. **(B)** BRCA1 knockdown in A549 cells makes the cells resistant to paclitaxel. In this field of view, 14 cells responded (27%) to the treatment and their MT cytoskeleton formed round bundles, 30 cells were not affected (58%) by the drug and did not form bundles at all, 4 cells were partially affected (7.5%) but did not form clear bundles, and 4 cells were already apoptotic (7.5%) by the time of imaging.

**FIGURE 9:**
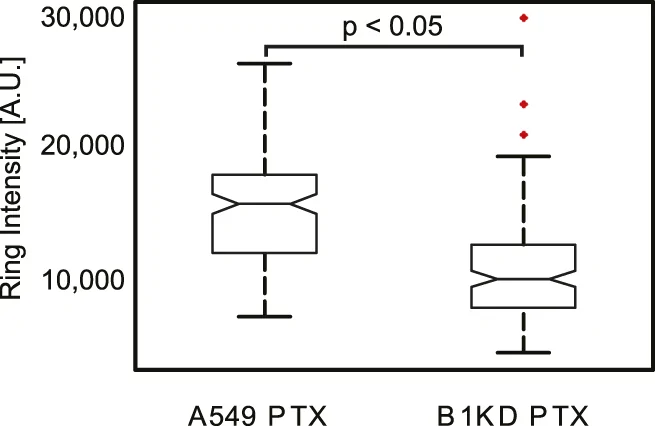
Quantification of paclitaxel (PTX) resistance resulting from knockdown of BRCA1 (B1KD) in adenocarcinoma (A549) cells. Cells were treated with 100 nM paclitaxel for 18 h. Cells that were sensitive to the drug, formed circular tubulin bundles after treatment (see Figure 8A for the 34 drug (83%) sensitive cells in the parental A549 cell line and Figure 8B for the 14 (27%) sensitive cells in the B1KD cell line). Comparison of the overall pixel intensity of these tubulin rings formed after paclitaxel treatment indicates much lower drug response with fewer bundles formed by stabilized MTs in the B1KD cell line, for which the overall ring intensity was lower with about 7,000 (17,000 vs. 10,000) [A.U., arbitrary units] as a median value. The differential drug-induced effects in the sensitive cells of the two cell lines suggest an additional mechanism of taxane resistance and a partial drug response in the B1KD cells.

In drug-sensitive interphase cells, MT bundle formation is the result of drug-mediated MT polymer stabilization and as such it represents a hallmark of taxane cellular activity (Sung and Giannakakou, 2014). MT bundles eventually form circular shapes as cells approach apoptosis. We applied a circular Hough transform to detect MT bundling and reorganization in drug-treated cells (Figure 8). Hough transform is a computational technique which allows the automated detection of a variety of shapes in images (Hough, 1959), most commonly used in the area of computer vision for line detection (Mattavelli et al., 2001). It transforms image space into parameter space, enabling the detection of shapes through pattern identification in the transformed space. Such algorithms have been in use, for instance, by the Danish postal services since the onset of the century to sort mail by automatically detecting the envelope stamp and have drastically improved the sorting efficiency, and decreased the utilization of manual labor. Several hours after treatment with paclitaxel, A549 cells began changing their shape into a circle and formed thick tubulin rings in 83% of the cells (Figure 8A), which indicates sensitivity to drug action. Knockdown of the BRCA1 gene in A549 cells (Sung and Giannakakou, 2014) affects their susceptibility to treatment with paclitaxel and they become resistant (Figure 8B). There is a variability in the cellular response in terms of morphological changes and only 27% changed their shape into a circle and formed thick tubulin rings (Figure 8B). Further, when we compared the pixel intensity over these rings of bundled MTs in the affected cells from the parental A549 cells and the derivative BRCA1 knockdown cells, the overall brightness was 70% higher in the paclitaxel-susceptible parental cells (Figure 9). These results indicate that even if there are measurable drug-induced changes in about a quarter (27%) of the resistant derivative BRCA1 knockdown cells, the effects of paclitaxel are significantly reduced. Overall, these data demonstrate two different mechanisms of taxane resistance.

#### Correlation of live-cell MT dynamics and gene expression signatures

To validate any *ex vivo* results regarding drug response and resistance, one could correlate the MT dynamics analysis in patient-derived tumor organoids with gene expression data and clinical response of the same patients. Our objective has been to identify candidate targets amongst the proteins that regulate particular parameters of MT dynamics (Lyle et al., 2009a; Lyle et al., 2009b). To achieve this end, we will use pattern analysis algorithms (He et al., 2013; Tiemann et al., 2013) and expect to be able to match the patient gene expression alterations (Supplementary Figure S3) to the pattern of deregulation of MT dynamics in a particular tumor (Matov, 2024e). Having measured MT deregulation patterns across multiple patients, we will create profiles of susceptibility to tubulin inhibitors within a statistical framework that classifies MT trajectories by mapping gene expression patterns to patient treatment outcomes (similar to (Monnier et al., 2015)).

#### Validation of epigenetic signatures and evaluation of therapeutic interactions

**FIGURE 10:**
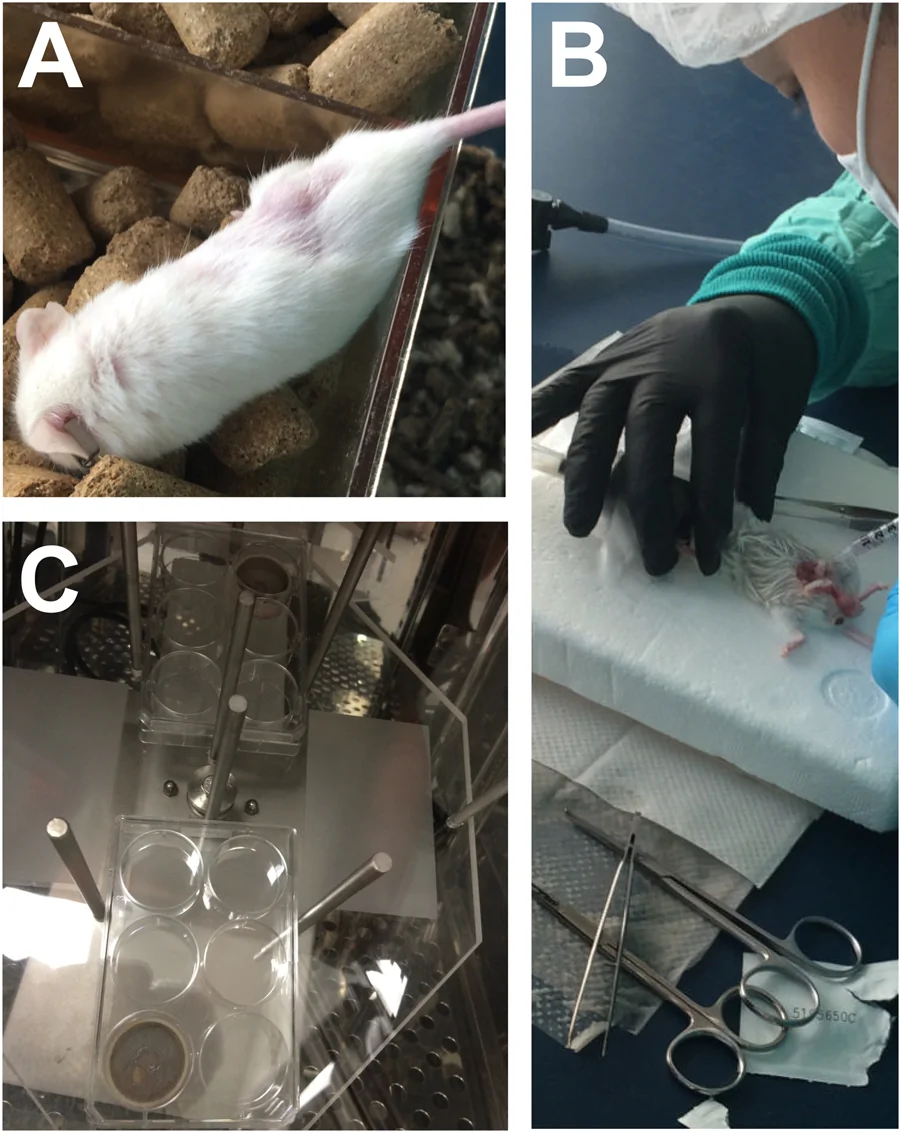
Injecting PC organoids to establish patient-derived xenografts in PC and culturing PC tissue *ex vivo*. **(A)** Tumor tissue from a radical prostatectomy case was planted subcutaneously by a surgeon in an immunodeficient mouse. **(B)** Injecting metastatic PC organoids into an immunodeficient mouse. **(C)** Culturing PC tumor tissue on a (bioreactor) shaker. Upper right and lower left wells contain slices of tissue from 1 mg punch core biopsy with Gleason Score 5 + 4 and 80% tumor density to evaluate drug action by computing the changes in the power spectrum of tissue as a 3D hologram (Jeong et al., 2010) after treatment with tubulin inhibitors.

Next, as MT-regulated genes implicated in drug resistance for the concrete patient are identified, organoids from knockdown experiments of inhibiting multiple of these MT regulators could be injected into mice and tested for an improved drug response (Figure 10). We will test knockdown organoids in terms of MT dynamics and dose-response morphology with image texture metrics (Matov, 2024b) as well as for alteration in drug susceptibility in patient-derived xenograft models in this context. We tested culturing of prostate tumor tissue slices for up to 48 h (Keshari et al., 2013) without tissue dissociation using a bioreactor (Maund et al., 2014) with the objective of developing another assay for the evaluation of drug action by computing and comparing the changes in the power spectrum of tissues after drug treatment imaged as a 3D hologram (Jeong et al., 2010). Another validation approach is to compare organoid gene expression signatures via high-throughput transcriptomics, which can evaluate the pharmacogenomics and establish biological pathway-altering concentrations (Harrill et al., 2021). By utilizing multiple validation methods, we will be able to establish not only the ability of our approach to anticipate and overcome drug resistance but also to evaluate whether the use of putative compounds will induce accumulative side effects beyond the desired metabolic changes for drug efficacy (Phillips et al., 2018).

## Discussion

### A specialized culture system and optimized media formulation

The most common metastatic site in PC is bone (84%) and liver is the most commonly the secondary site (39%) (Gandaglia et al., 2014). Neither organoid culture nor murine models provide the required environment for patient cells from bone metastasis. To provide a more suitable environment, we propose to culture cells derived from bone biopsy on hFOB 1.19 cells, which mimic the human bone, in a specialized microfluidics device (Matov, 2024b; Zhang et al., 2015). The bone marrow culture medium consists of RPMI with L-glutamine, patient plasma, CaCl_2_, sodium succinate, hydrocortisone, heparin (Zhang et al., 2015), and one should allow for 48 h of metabolic recovery after the biopsy before an *ex vivo* drug treatment. After plating patient cells in such microfluidics, about 70% of them survive (Zhang et al., 2014). To further increase the rates of patient cell survival after plating, additional media components could be added both in the context of mimicking the bone marrow (Aleman et al., 2019) and improving fibroblast activation (Matov, 2025a; Tran et al., 2012).

### Impact of modulating MT dynamics in immunotherapy

About one-third of the patients progressing after treatment with hormonal therapies have intrinsic resistance to systemic therapy. The two-thirds who respond, suffer visible side effects from drug toxicity and rapidly acquire resistance to therapy. At present, clinical data to delineate properly the differences between the taxanes is still missing, that is, one can identify and quantify differences in computational metrics from research point of view, but the clinical angle is limited to cabazitaxel being a more potent version of docetaxel. Novel tubulin inhibitors will be introduced in the clinic and used at earlier stages of disease progression and once this is done, better links between clinical outcomes and research will be established. Overall, the treatment of advanced PC is hampered by the lack of known molecular markers for reliable targeted therapies. In this contribution, we have proposed methods to anticipate drug resistance in patient cells *ex vivo* and inform therapy. We have also discussed an approach for sensitizing resistant prostate tumors by targeting the tubulin transcriptome. Further, checkpoint blockade inhibitors provide responses of unprecedented durability for a fraction of patients with otherwise treatment-refractory solid tumors (Pardoll, 2012) and will become the standard of care (Topalian and Pardoll, 2025). Our hypothesis is that tumors of the prostate, of which only a small fraction were considered immunogenic (Shenderov et al., 2023; Wu et al., 2018), can be modulated with compounds that alter cytoskeletal regulation to become susceptible to immunotherapy.

**FIGURE 11:**
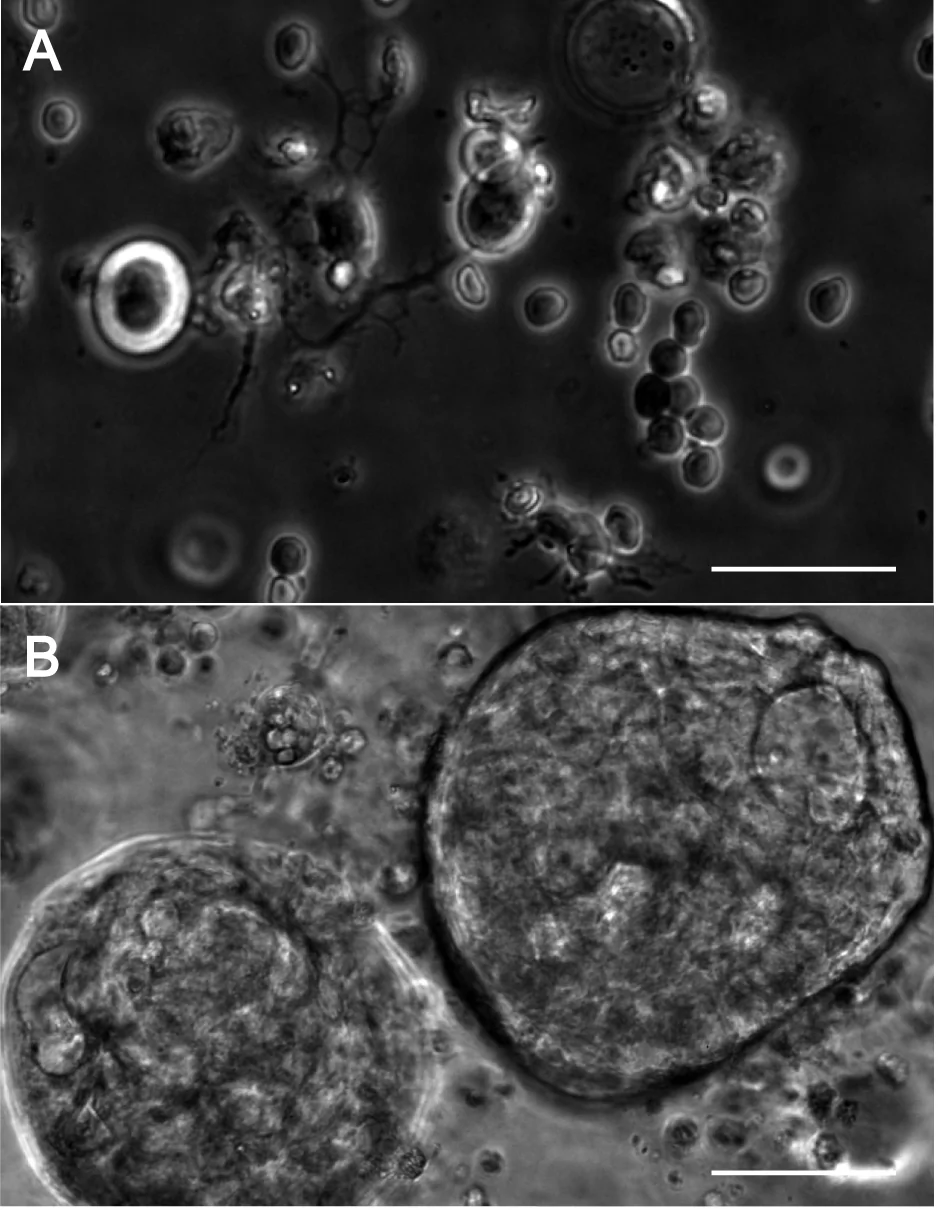
Prostate cancer organoids. Phase contrast microscopy. 20× magnification. **(A)** UCSF-PCa2 (metastatic tumor, visceral tissue (liver)) on Day 1. Tumor was obtained from ½ mg tissue from a needle biopsy. Lymphocytes are visible as well. Scale bar equals 80 µm. **(B)** UCSF-PR9 (primary tumor) on Day 20. Intraductal PC organoids obtained from organ-confined disease. Tumor was obtained from 1 mg tissue (Gleason Score 5 + 4, 80% tumor density, punch core biopsy) from a radical prostatectomy. Ducts are visible in the organoids. Scale bar equals 60 µm.

We observed patient tumor-infiltrating lymphocytes (TILs) in our organoid culture in the days after tissue dissociation of distant metastasis samples, whereas cultures derived from primary tumors formed organoids with specific shapes characteristic of the tissue of origin, such as the prostatic ducts (Figure 11). A tumor with a high number of TILs could have hundreds or thousands of T cells and other immune cells that are all tumor-specific (Zorko et al., 2024). However, the majority of patients experience primary resistance due to low TIL cell infiltration. In PC, a 40 mL peripheral blood draw would not contain a sufficient number of tumor-specific TIL cells and to make immunotherapy feasible, TILs can be isolated, cultured (starting by plating 10 tumor TIL cells per well), and expanded *ex vivo*. Next, a chemical screen can identify small molecule enhancers of TIL cell response in patient-derived tumor organoids. Small molecules can alter the immunogenicity of tumor cells and promote tumor-specific T-cell and natural killer (NK) cell activation (Mitchison, 2021). Drug candidates can be identified via an unbiased cell-based screen in a tumor organoid and an immune cell co-culture format utilizing patient-derived tumor and matched blood samples. Lytic granules from cytotoxic T cells have been shown to exhibit kinesin-dependent motility on MTs (Burkhardt et al., 1993). T-cell and NK cell activation can be assayed by high-throughput measurements of cytokine production and quantitative cell biological profiling (Matov, 2024h).

### Clinical relevance of MT dynamics in combination therapy

No one has been cured of metastatic PC. Patient tumors are treated with drugs that lower the androgen levels, which induces Alzheimer’s disease (Zhang et al., 2024). Even if approved only in the metastatic setting, androgen-targeting therapy is used off-label in some hospitals to treat primary tumors as well. Androgen deprivation initially lowers patient PSA levels and keeps the tumors at bay for a few years. The disease, once treated with androgen inhibitors, unfortunately, always returns in a very aggressive form. Slow-proliferating, heterogeneous prostate tumors then evolve to a fast-proliferating clonogenic metastatic disease for which there are very limited treatment options. Prostate tumor cells (unlike prostate cells derived from normal tissues) cannot be easily cultured *ex vivo* or transplanted in mouse models and that indicates the existence of a specific microenvironment in the human body in PC which is poorly understood. The fact, however, that more and more clinical studies recommend the utilization of tubulin inhibitors earlier in disease progression, including as neo-adjuvant therapy (Sumiyoshi et al., 2024), suggests an interplay between AR and MT regulation. GSK3 is a well-established component of the Wnt signaling pathway and one of the most prominent regulators of MT homeostasis (Frame and Cohen, 2001). This kinase sits in the signal transduction and phosphorylation cascade one level above the immediate MT-associated proteins and regulates their behavior (Hajka et al., 2021). GSK3 inhibitors are currently being tested for over 20 clinical indications from neurodegeneration to a variety of cancers (Duda et al., 2020; Wood, 2022). We hypothesize that targeting such genes, which are located at an earlier stage of signal transduction than the transcription factor AR, could bring the PSA levels to normal values without triggering a cascade of long-term side effects.

**FIGURE 12:**
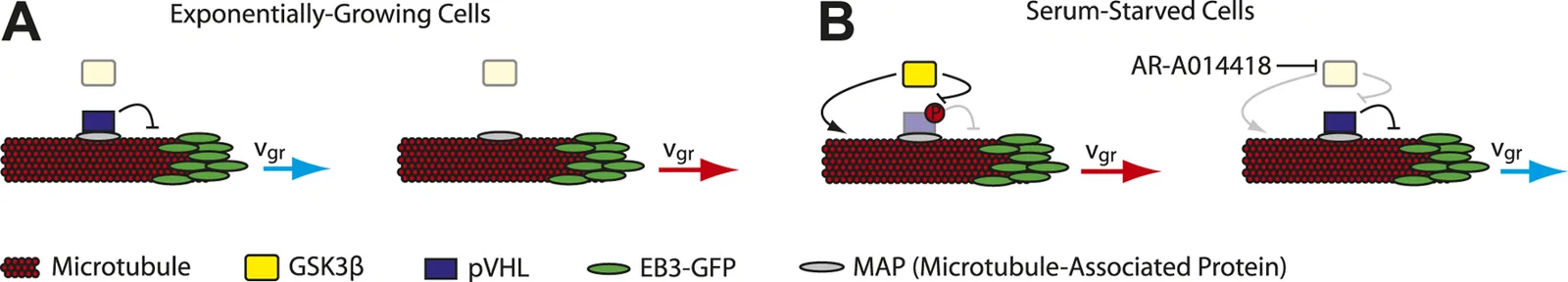
Loss of pVHL in exponentially-growing cells and GSK3β activity in quiescent cells lead to an increase in MT turnover rates. pVHL attenuates MT dynamics by lowering the rates (blue arrows) of tubulin turnover both at growing and shrinking MT tips, and by inhibition of MT catastrophe events, likely because pVHL30 reduces the intrinsic GTPase activity (Thoma et al., 2010). These functions of pVHL contribute to the changes in MT dynamics when cells are serum-starved and are negatively regulated by GSK3β. **(A)** In exponentially-growing cells with a functioning tumor suppressor protein pVHL, the loss of pVHL leads to an increase in the rates of MT polymerization (left red arrow). **(B)** In serum-starved (quiescent) cells, GSK3β phosphorylation of pVHL leads to fast tubulin turnover (right red arrow). The inhibition of GSK3 restores pVHL function and baseline MT dynamics. MT-associated proteins, like CLASP2, regulate MT dynamics as well.

In addition, different GSK3 isoforms are elevated in early (GSK3α) and advanced disease (GSK3β) (Duda et al., 2020), providing a rationale for differential targeting and giving hope that Gleason Score 3 tumors can be cured. Low-grade tumor cells maintain their progenitor cell attributes (Lukacs et al., 2008) and it is conceivable that lowering the levels of GSK3α (Ma et al., 2017) may revert the differentiation processes. MT dynamics can offer a functional assay to screen putative compounds that correct aberrant MT homeostasis in disease (Matov, 2024e), such as the increase of MT polymerization rates during interphase, which likely shortens interphase and accelerates cell cycle progression and mitotic entry in a subset of metastatic PC cells. These agents might be inhibitors of a variety of kinases (Kannaiyan and Mahadevan, 2018) and MT-associated proteins (Figure 12) (Čermák et al., 2020), or novel compounds that act on the DNA binding (Pang et al., 2022) and transactivation (Zhu et al., 2022) domains of AR. After validation and calibration, our software can be utilized for the discovery of novel drug combinations that allow personalized treatment of PC.

### Drug dose selection and drug development

When a drug regimen is selected, the selection of drug dose and deducing its effects (Zasadil et al., 2014), depending on the dose administered, is a daunting task. During treatment with tubulin inhibitors, for instance, different MT-associated proteins are activated depending on the drug dose administered, and consequently, different resistance mechanisms may be triggered (Matov, 2024e; Matov, 2024f). The effects of MT inhibitors change nonlinearly with a dose increase. While a high dose of nocodazole depolymerizes MTs, a low dose, which could lead to no side effects, increases polymerization rates (Vid. 6) (Matov, 2024e; Thoma et al., 2010) and that would induce different medical effects. While a high dose of paclitaxel stabilizes MTs, a low dose similarly increases MT polymerization rates, which can, counterproductively, assist cancer cells in progressing through mitosis (Ertych et al., 2014; Kuo et al., 2025; Matov, 2024d; Matov, 2024e).

During pathogenesis, or as a result of drug treatment in disease (Gonçalves et al., 2001), cells change their intracellular organization (Galletti et al., 2014; Matov, 2024e), rearrange their internal components as they grow, divide, and adapt mechanically to a hostile environment. These functions depend on the controlled polymer turnover of protein filaments (the cytoskeleton and FAs), which provide the cell shape (Matov, 2024b) and its capacity for directed movement (Lauffenburger and Horwitz, 1996) and, thus, determine tumor aggressiveness and sensitivity to drug treatment. In this context, specialized image analysis approaches (Matov, 2024h) for the quantification of the physiological intercellular protein dynamics (Matov, 2024h) in patient-derived cells might become an indispensable tool for drug development as well as the selection of optimal drug regimens (Matov, 2025a) and drug dose selection in both organ-confined and metastatic PC.

## Materials and methods

### Coverslip processing

Peripheral blood previously drawn from mCRPC patients (the samples were de-identified) receiving docetaxel-based first-line chemotherapy was thawed and processed using ficolling (Ficoll-Paque®) to separate cells in the buffy coat from the blood. The cells were isolated by centrifugation, fixed in phenol, and blocked on 8 mm coverslips. In brief, the ficolling method, through centrifugation, isolates peripheral blood mononuclear cells and plasma from granulocytes and erythrocytes. The mononuclear layer contains lymphocytes, monocytes, and CTCs. These cells are plated onto an 8 mm coverslip and subsequently stained for biomarkers (PSMA, CD45, and DAPI). PSMA labeling was done using ^177^Lu -J591, anti-PSMA monoclonal antibody J591 (Matov, 2024g).

### Cell culture

Primary and retroperitoneal lymph node metastatic prostate tumor and sternum metastatic rectal tumor tissues were dissociated to single cells using modified protocols from the Witte lab (Goldstein et al., 2011). Organoids were seeded as single cells in three 30 µL Matrigel drops in 6-well plates. Organoid medium was prepared according to modified protocols from the Clevers lab (van de Wetering et al., 2015) and the Chen lab (Gao et al., 2014).

**FIGURE 13:**
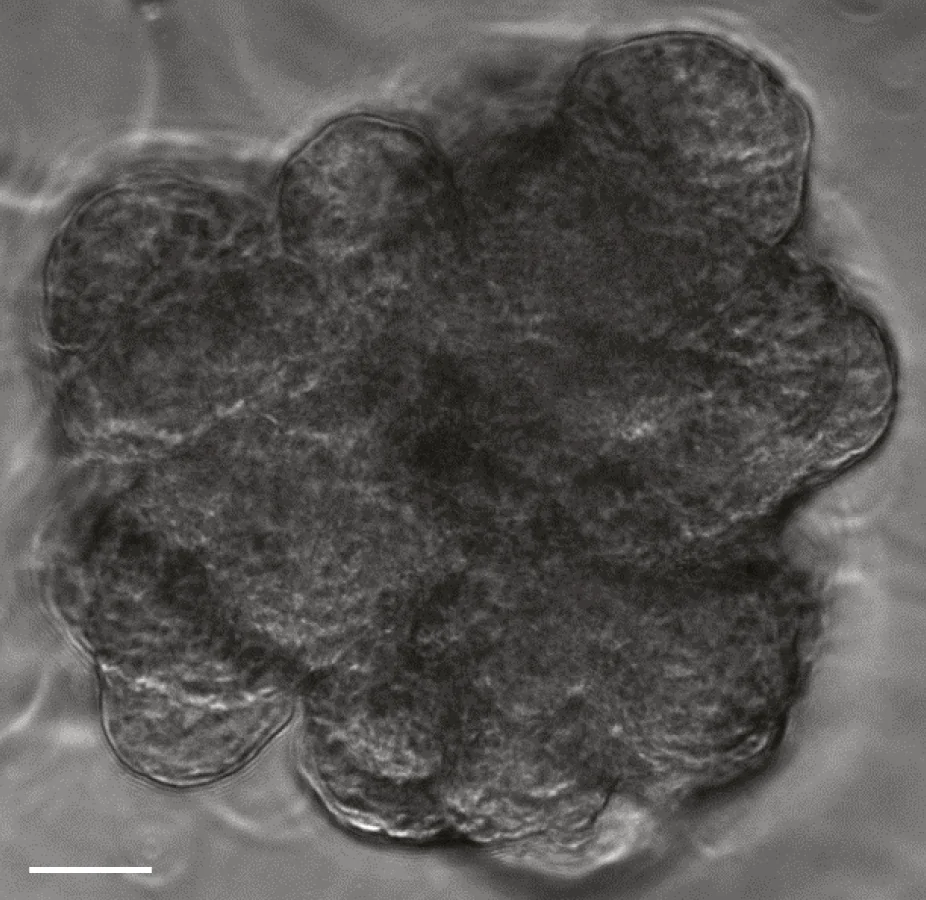
Phase contrast microscopy image of a breast cancer organoid. Organoids derived from CTCs from a mouse xenograft model of invasive ductal breast carcinoma. Scale bar equals 30 µm.

To obtain a single-cell suspension, tissues were mechanically disrupted and digested with 5 mg/mL collagenase in advanced DMEM/F12 tissue culture medium for several hours (between 2 and 12 h, depending on the biopsy or resection performed). If this step yielded too much contamination with non-epithelial cells, for instance during processing of primary prostate tumors, the protocol incorporated additional washes and red blood cell lysis (Goldstein et al., 2011). Single cells were then counted using a hemocytometer to estimate the number of tumor cells in the sample, and seeded in growth factor-reduced Matrigel drops overlaid with PC medium (Gao et al., 2014). With radical prostatectomy specimens, we had good success with seeding 3,000 cells per 30 μL Matrigel drop, but for metastatic samples organoid seeding could reliably be accomplished with significantly fewer cells, in the hundreds. To derive organoids from patient CTCs, liquid biopsy samples of 40 mL peripheral blood would be collected, processed, and plated in a Matrigel-collagen-fibronectin matrix to form organoids similarly to the metastatic breast cancer organoids we cultured from mouse CTCs (Figure 13).

To transduce organoids, we modified protocols from the Clevers lab (Koo et al., 2013) to adapt to the specifics of prostate organoid culture (such as the significant differences in proliferation rates in comparison to colon and rectal organoids). We found out empirically that cells in mid-size organoids (60-100 µm in diameter) infect at much better rates than trypsinized single organoid cells, small organoids, or large organoids. The steps we followed to express EB1ΔC-2xEGFP in organoids were: (1) Add dispase (1 mg/mL) to each well to dissolve the Matrigel at room temperature for 1 h. (2) Spin down (at 1,000 rpm for 4 min) and mix organoids with 10 µL of viral particles (enough for 1 well with three Matrigel drops of 30-40 µL with organoids containing 1 to 2 million cells), 10 mg/mL Y27632 ROCK inhibitor, and 10 mg/mL polybrene for 30 min. (3) Spin the organoids with viral particles for 1 h at 600 g. (4) Leave the organoids for a 6-h incubation. (5) Spin down and plate in Matrigel. (6) 1 h later, add medium. We used 0.5 mg/mL blasticidin for only one round of medium (3 days) because an increase in the density of labeled cells in the organoids reduced our ability to image MT tips with good contrast.

### Live-cell MT tracking

M12 cells were transfected with plasmid encoding EB1ΔC-2xEGFP (Piehl and Cassimeris, 2003) and EB1 comets were tracked using our image analysis algorithm (Matov et al., 2010). EB1 transiently binds to growing MT plus tips (Akhmanova and Steinmetz, 2008), generating a punctate pattern of EB1ΔC-2xEGFP comets throughout the cell. The exponential decay of available binding sites results in the characteristic comet-like fluorescence profiles of EGFP-tagged end-binding proteins (“comets”). MDA-MB-231 cells were transiently transfected with EB1ΔC-2xEGFP and treated with vehicle (DMSO) or treated with 100 nM paclitaxel for 2 h. Paclitaxel (in intravenous solution) was a gift from Linda Vahdat. To test for statistically significant shifts in the distributions of MT growth rates obtained following the tracking of 195,970 EB1ΔC-2xEGFP comets, we performed per-MT track and per-cell analysis, and compared the average EB1 comet speeds for 14 DMSO-treated and 14 paclitaxel-treated cells (see Statistical Analysis). Clear cell renal cell carcinoma (ccRCC) cells were cultured and imaged as previously described (Thoma et al., 2010). Receptor triple-negative breast cancer (MDA-MB-231) cells were cultured and imaged as previously described (Matov, 2024e).

### Microscopy imaging

The type of microscopy used to image fixed cells was spinning-disc confocal microscopy with a 10× magnification 0.3 NA objective. The acquisition was accomplished using an ORCA-Flash 4.0 (6.5 µm/pixel) camera. EB1 comets in live cells were imaged by spinning-disc confocal microscopy using a 100× magnification oil immersion 1.49 NA objective for all cultured cells as previously described (Gierke and Wittmann, 2012). We imaged EB1ΔC-2xEGFP-expressing organoids (Koo et al., 2013) by time-lapse spinning disk confocal microscopy using a long working distance 60× magnification water immersion 1.45 NA objective. We acquired images at half a second intervals for a minute to collect datasets without photobleaching (Gierke and Wittmann, 2012).

### Image analysis

All image analysis programs for drug-induced tubulin bundling, EB1/3 comet detection, MT dynamics, and graphical representation of the results were developed in Matlab and C/C++. The computer code is available for download at: https://github.com/amatov/ClusterTrackTubuline and https://github.com/amatov/ResistanceBiomarkerAnalysis.

### Statistical analysis

Statistical tests were performed in Matlab. We performed Kolmogorov-Smirnoff statistics (Kolmogorov, 1933) to compare higher-order moments of the underlying MT parameter per-track distributions and permutation *t*-test to extract information on shifts in median values of polymerization parameters over a population of cells. In brief, for each condition, multiple videos containing one or two cells were compared. Before merging the data for further statistical analysis, we determined the video-to-video variation in the distributions of MT parameters using the Kolmogorov-Smirnov test (Kolmogorov, 1933). Distributions were subsampled to 400 values to avoid hypersensitivity of the Kolmogorov-Smirnov statistics. Videos passing the consistency test were included in the final datasets. Statistical comparison of MT dynamics between different conditions was accomplished in two steps. First, we examined whether the different conditions would profoundly change the distribution of each parameter, which would indicate an entirely different state of MT regulation between conditions. To test this possibility, we aligned the distributions of compared conditions at their respective per-track mean value and then applied the nonparametric Kolmogorov-Smirnov test to determine whether the two distributions are identical. The distributions were randomly subsampled to 400 values. The subsampling was repeated 200 times, and the final p-values were derived by averaging. In a second step, we compared the shifts in parameter mean values between different conditions. To achieve this, we calculated the per-cell means for each parameter. Then we tested the normality of the distribution of the means and applied a test to define the significance of parameter changes between conditions (mean ± SEM of cell-to-cell variation). This test assumes that the cell-to-cell variation is the single biggest source of variation in the mean value of a condition. None of the parameters of MT dynamics followed a Gaussian distribution. To determine the significance of differences in the mean values between parameters under different experimental conditions, we applied a non-parametric permutation *t*-test with 400 repetitions (Hesterberg et al., 2005) which compares the bootstrapped distributions of condition 1 versus condition 2. This test does not make any assumptions about the characteristics of the tested distribution and, thus, is appropriate for application to distributions that are far from being normally distributed. In brief, for each condition 400 values are bootstrap-sampled from the data. In agreement with the central limit theorem (Laplace, 1810), the two bootstrapped distributions always follow a Gaussian distribution and, thus, could be analyzed for differences using a regular Student’s *t*-test.

## Data Availability

The original contributions presented in the study are included in the article/Supplementary Material, further inquiries can be directed to the corresponding author.

## Glossary

ADT: androgen depravation therapy
AR: androgen receptor (AR-wt – wild type, ARv567 and AR-V7 – splice variants of the AR)
BRCA1: breast cancer type 1 susceptibility protein
CLASP2: cytoplasmic linker-associated protein, 2
CLIP-170: cytoplasmic linker protein, 170
CTC: circulating tumor cell
DMSO: dimethyl sulfoxide
EB1/3: microtubule end-binding protein, 1/3
EB1ΔC: EB1 construct truncated at amino acid 248 that does not interact with other proteins at the microtubule end
EGFP: enhanced green fluorescent protein (2xEGFP indicates two molecules)
ERG: erythroblast transformation-specific family-related gene
FGF: fibroblast growth factor
GPX4: glutathione peroxidase, 4
GSK3α/β: glycogen synthase kinase, 3, alpha/beta isoforms
HIF1α: hypoxia-inducible factor, 1, alpha subunit
MAPK: mitogen-activated protein kinase
MT: microtubule
NAD^+^/NADH: nicotinamide adenine dinucleotide, oxidized/reduced form
NK: natural killer cell
Op18: oncoprotein, 18 (stathmin)
PC: prostate cancer (mCRPC–metastatic castrate-resistant prostate cancer)
PSMA: prostate-specific membrane antigen
ROCK: Rho-associated coiled-coil kinase
TMPRSS2: transmembrane protease, serine, 2
pVHL: von Hippel-Lindau protein (pVHL30/pVHL19 refer to the long/short isoform)
