# Supplementary material for: Computational analysis of treatment resistant cancer cells
Source: Front Cell Dev Biol. 2026 Aug 25;13:1723251. doi: 10.3389/fcell.2025.1723251 (PMC13552209; doi:10.3389/fcell.2025.1723251)
Supplement: Supplementary file 1 [file Supplementaryfile1.doc]

**SUPPLEMENTARY MATERIALS**

Figure S1 – Tracking of EB1 comets in M12 parental androgen-independent cell line.

Figure S2 – CTC with diameter larger than that of a normal prostate epithelial cell.

Figure S3 – Expression signatures of 70 MT regulating genes for MSKCC patient organoids.

Figure S4 – Tracking of EB1 comets in MCF10A normal breast epithelium cell line.

Video 1 – PC organoid (MSK-PCa5) derived from CTCs with labeled MT tips and overlaid EB1 comet detections. The organoid was established at MSKCC from CTCs of a mCRPC patient with a high CTC count (>100 cells per 8 mL of blood) (Gao et al., 2014). MT tips were labeled, imaged, and computationally detected (green dots in the video; the cell diameter is 10 μm) as visible EB1ΔC-2xEGFP comets (Matov et al., 2010).

Video 2 – RCC-4 VHL^-/-^ nocodazole 40nM EB3ΔC-2xEGFP comet detections. Detection of visible EB3ΔC-2xEGFP comets (Matov et al., 2010) 20 seconds after the addition of nocodazole.

Video 3 – RPE-1 ns-control EB3ΔC-2xEGFP (Thoma et al., 2010) with overlaid clusters of collinear MT tracks (Matov et al., 2010). Visible stages of MT growth are shown in white color, forward gaps (stages of MT pausing) are shown in green color, and backward gaps (stages of MT shrinking) are shown in red color.

Video 4 – RPE-1 *shVHL* EB3ΔC-2xEGFP with overlaid clusters of collinear MT tracks (Matov et al., 2010). Visible stages of MT growth are shown in white color, forward gaps (stages of MT pausing) are shown in green color, and backward gaps (stages of MT shrinking) are shown in red color.

Video 5 – RPE-1 ns-control CLIP170-2xEGFP comet detections (Matov et al., 2010).

Video 6 – RCC-4 pVHL30 nocodazole 40nM EB3ΔC-2xEGFP comet detections. Detection of visible EB3ΔC-2xEGFP comets (Matov et al., 2010) 20 seconds after the addition of nocodazole.

The movies have a length of 12.5 seconds. Original time-lapse sequences consist of 125 frames acquired with a frame rate of 0.5 seconds. The replay rate of the movies is 10 frames per second, that is, a five-fold acceleration.

**Figure S1. Tracking of EB1 comets in M12 parental androgen-independent cell line.** (A) Detection of growing MT tips (Matov et al., 2010) indicates high density of polymerizing MTs and EB1 comets, whose center of mass is displayed with a green dot. (B) MT tips are labeled with GFP, imaged for a minute (with an acquisition rate of two images per second), and EB1 comets are computationally tracked (Yang et al., 2005). The average MT growth rates and EB1 speeds in parental M12 cells not expressing AR is 17.3 μm/min, which is reduced with about 1 μm/min when AR-wt is expressed and increased with over 7 μm/min when the taxane-resistant Arv7 variant is expressed. The color-coding represents EB1 speeds; colder colors correspond to lower speeds, and warmer colors correspond to faster speeds. The scale bar equals 5 µm.

**Figure S2. CTC with diameter larger than that of a normal prostate epithelial cell.** Multiplex, 10x magnification, transmitted light microscopy images of a patient blood sample (Matov, 2024b). One CTC and two leukocytes are visible in this field of view. (A) PSMA (J591, tumor marker). (B) CD45 (leukocyte marker). (C) DAPI (nuclear marker). The scale bar equals 20 µm.

**Figure S3. Gene expression signatures of 70 MT regulating genes for MSKCC patient organoids.** GSK3 regulates most of the MT regulating genes (Hajka et al., 2021). Hierarchical clustering results indicate that STMN1/Op18 is upregulated in MSK-PCa1, for which we measured attenuated MT polymerization (growth) rates (Matov, 2024a). MT growth rates [µm/min] in cells from organoids PCa1, PCa2, PCa3, and PCa5 are regulated by multiple genes directly, such as chTOG (CKAP5/XMAP215), MAP2, MAP4, CLIP-170, and indirectly by other, such as Op18, TPX2 (Lyle et al., 2009a; Lyle et al., 2009b). MT polymerization times [seconds] are reversely correlated with MT catastrophe frequency, that is, the longer the lifetime of measured EB1 track are, the less likely it is MTs to switch to state of shrinking. MT depolymerization rates [µm/min] reflect the speed with which MTs remove dimers from their lattice during shrinking. The regulation of stages of MT depolymerization is not well understood. MT depolymerization times [seconds] are reversely correlated with the MT rescue frequency, that is, the longer MTs are shrinking, the less likely it is MTs to switch to state of growth. Pausing times [seconds] reflect the moments MTs neither grow nor shrink. Our work on the pVHL tumor suppressor protein demonstrated that pausing times are significantly increased when the tumor-suppressor protein is active and reduces the effects of the MT-depolymerizing drug nocodazole (Thoma et al., 2010).

**Figure S4. Tracking of EB1 comets in MCF10A normal breast epithelium cell line.** (A) MT tips are labeled with GFP, imaged for a minute (with an acquisition rate of two images per second), and EB1 comets are computationally tracked (Yang et al., 2005). The color-coding represents EB1 speeds; colder colors correspond to lower speeds, and warmer colors correspond to faster speeds. The scale bar equals 5 µm. Histogram of MT growth rates in µm/min is shown on (B) and indicates a normal distribution and suggests that the EB1 speeds (with a median value of about 20 µm/min and an average of 18.7 µm/min), resulting from a faster MT polymerization in normal breast epithelium, are about 30% to 70% faster (79,560 EB1 comets in MCF10A cells were analyzed) in comparison to breast cancer cells (see Fig. 6 and (Matov, 2024a)). Nicotinamide adenine dinucleotide (NAD^+^) is an endogenous small molecule that has effects on diverse processes, including obesity, lifespan, and cancer, which can increase MT polymerization rates (Harkcom et al., 2014) and restore normal MT regulation and homeostasis.

Gao, D., I. Vela, A. Sboner, P.J. Iaquinta, W.R. Karthaus, A. Gopalan, C. Dowling, J.N. Wanjala, E.A. Undvall, V.K. Arora, J. Wongvipat, M. Kossai, S. Ramazanoglu, L.P. Barboza, W. Di, Z. Cao, Q.F. Zhang, I. Sirota, L. Ran, T.Y. MacDonald, H. Beltran, J.M. Mosquera, K.A. Touijer, P.T. Scardino, V.P. Laudone, K.R. Curtis, D.E. Rathkopf, M.J. Morris, D.C. Danila, S.F. Slovin, S.B. Solomon, J.A. Eastham, P. Chi, B. Carver, M.A. Rubin, H.I. Scher, H. Clevers, C.L. Sawyers, and Y. Chen. 2014. Organoid cultures derived from patients with advanced prostate cancer. *Cell*. 159:176-187.

Hajka, D., B. Budziak, Ł. Pietras, P. Duda, J.A. McCubrey, and A. Gizak. 2021. GSK3 as a Regulator of Cytoskeleton Architecture: Consequences for Health and Disease. *Cells*. 10.

Harkcom, W.T., A.K. Ghosh, M.S. Sung, A. Matov, K.D. Brown, P. Giannakakou, and S.R. Jaffrey. 2014. NAD+ and SIRT3 control microtubule dynamics and reduce susceptibility to antimicrotubule agents. *Proc Natl Acad Sci U S A*. 111:E2443-2452.

Lyle, K., P. Kumar, and T. Wittmann. 2009a. SnapShot: Microtubule Regulators I. *Cell*. 136:380, 380.e381.

Lyle, K., P. Kumar, and T. Wittmann. 2009b. SnapShot: Microtubule regulators II. *Cell*. 136:566, 566.e561.

Matov, A. 2024a. Mitosis, Cytoskeleton Regulation, and Drug Resistance in Receptor Triple-Negative Breast Cancer. *arXiv*:2407.19112.

Matov, A. 2024b. Quantitative Microscopy in Medicine. *medRxiv*:2024.24311304.

Matov, A., K. Applegate, P. Kumar, C. Thoma, W. Krek, G. Danuser, and T. Wittmann. 2010. Analysis of microtubule dynamic instability using a plus-end growth marker. *Nature Methods*. 7:761- 768.

Thoma, C.R., A. Matov, K.L. Gutbrodt, K.R. Hoerner, W. Krek, and G. Danuser. 2010. Quantitative image analysis identifies pVHL as a key regulator of microtubule dynamic instability. *Journal of Cell Biology.* 190:991-1003.

Yang, G., A. Matov, and G. Danuser. 2005. Reliable tracking of large scale dense antiparallel particle motion for fluorescence live cell imaging. *In* Proc. IEEE Computer Vision and Pattern Recognition, San Diego. 138-146.
